# Mutual Inactivation of Notch Receptors and Ligands Facilitates Developmental Patterning

**DOI:** 10.1371/journal.pcbi.1002069

**Published:** 2011-06-09

**Authors:** David Sprinzak, Amit Lakhanpal, Lauren LeBon, Jordi Garcia-Ojalvo, Michael B. Elowitz

**Affiliations:** 1 Howard Hughes Medical Institute, California Institute of Technology, Pasadena, California, United States of America; 2 Division of Biology and Department of Applied Physics, California Institute of Technology, Pasadena, California, United States of America; 3 Departament de Física i Enginyeria Nuclear, Universitat Politècnica de Catalunya, Barcelona, Spain; Ecole Normale Supérieure, France

## Abstract

Developmental patterning requires juxtacrine signaling in order to tightly coordinate the fates of neighboring cells. Recent work has shown that Notch and Delta, the canonical metazoan juxtacrine signaling receptor and ligand, mutually inactivate each other in the same cell. This *cis-*interaction generates mutually exclusive sending and receiving states in individual cells. It generally remains unclear, however, how this mutual inactivation and the resulting switching behavior can impact developmental patterning circuits. Here we address this question using mathematical modeling in the context of two canonical pattern formation processes: boundary formation and lateral inhibition. For boundary formation, in a model motivated by *Drosophila* wing vein patterning, we find that mutual inactivation allows sharp boundary formation across a broader range of parameters than models lacking mutual inactivation. This model with mutual inactivation also exhibits robustness to correlated gene expression perturbations. For lateral inhibition, we find that mutual inactivation speeds up patterning dynamics, relieves the need for cooperative regulatory interactions, and expands the range of parameter values that permit pattern formation, compared to canonical models. Furthermore, mutual inactivation enables a simple lateral inhibition circuit architecture which requires only a single downstream regulatory step. Both model systems show how mutual inactivation can facilitate robust fine-grained patterning processes that would be difficult to implement without it, by encoding a difference-promoting feedback within the signaling system itself. Together, these results provide a framework for analysis of more complex Notch-dependent developmental systems.

## Author Summary

Multicellular development requires tightly regulated spatial pattern formation, frequently including the generation of sharp differences over short length scales. Classic examples include boundary formation in the *Drosophila* wing veins and lateral inhibition patterning in the differentiation of sensory cells. These processes and a diverse variety of others are mediated by the Notch signaling system which allows neighboring cells to exchange information, via interaction between the Notch receptor on one cell and its ligands such as Delta, on another. Interestingly, recent evidence has shown that Notch and Delta within the same cell (in *cis*) also interact, mutually inactivating each other. However, the significance of this interaction for pattern formation has remained unclear. Here we show, by analytical and computational modeling, how this *cis* interaction intrinsically generates a difference-promoting logic that optimizes the system for use in fine-grained pattern formation. Specifically, boundary formation and lateral inhibition patterning both operate more effectively and with simpler circuit architectures than they could without this interaction. Our results provide a foundation for understanding these and other Notch-dependent pattern formation processes.

## Introduction

Notch signaling is the canonical metazoan juxtacrine signaling pathway. It is involved in many developmental processes in which neighboring cells adopt distinct fates. Examples of such processes include the delineation of sharp boundaries during the formation of *Drosophila* wing veins [1], [2] and the formation of checkerboard-like patterns of differentiation, as occurs during *Drosophila* microchaete bristle patterning [3].

Notch signaling occurs through contact between a Notch receptor on one cell and a Delta/Serrate/LAG-2 (DSL) ligand such as Delta or Serrate (Jagged in mammalian cells) on a neighboring cell. This interaction leads to cleavage of Notch, releasing its intracellular domain, which translocates to the nucleus and serves as a co-transcription factor to activate target genes [4]. In addition to this activating *trans* interaction between Notch and DSL on neighboring cells, inhibitory *cis* interactions between Notch and DSL in the same cell suppress Notch signaling [5], [6], [7], [8], [9], [10]. Recent work indicates that this *cis*-interaction between Notch and DSL is symmetric: Notch inhibits its ligand, and the ligand inhibits Notch [9], [11], [12]. The molecular mechanism of this mutual inactivation between Notch and DSL, and whether or not it occurs at the cell surface, is still unclear [9], [12], [13], [14].

**Figure 1:**
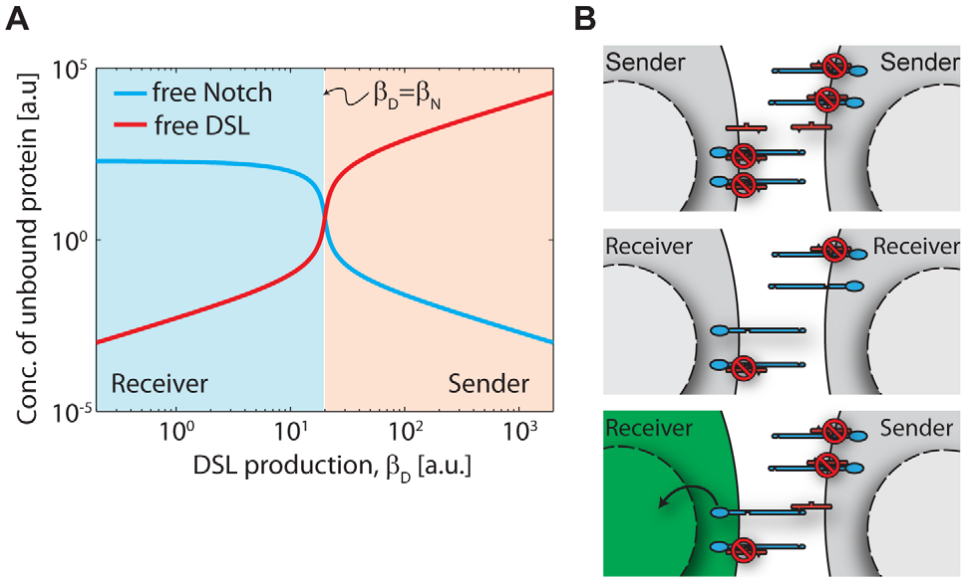
Ultrasensitivity due to mutual inactivation of Notch and DSL. (A) Plot of free DSL (red) and free Notch (blue) as a function of DSL production rate, 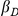. A sharp switch (high logarithmic derivative) between sender and receiver states occurs when 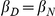. (B) Schematic illustration of sending and receiving states, showing that while very little signaling occurs when two neighboring cells are both senders (top) or both receivers (middle), strongly biased signaling can occur for the case of neighboring sender and receiver cells (bottom).

In an individual cell, mutual inactivation of Notch and DSL results in an ultrasensitive switch between ‘sending’ (low Notch/high DSL) and ‘receiving’ (low DSL/high Notch) cellular states (see Fig. 1) [11]. A cell with more total Notch than DSL (i.e. with a higher production rate of Notch than DSL given equal first order degradation rates) has an excess of free Notch but very little free DSL, making it a receiver (Fig. 1A, left). Conversely, a cell with more total DSL than Notch would have an excess of DSL and very little Notch, thus becoming a sender (Fig. 1A, right). In either state, both ligand-mediated inhibition of receptor and receptor-mediated inhibition of ligand contribute to the nonlinearity of the system. For a sufficiently strong *cis* interaction, the transition between these two states becomes very sharp, or ultrasensitive (Fig. 1A). This switch generates strongly-biased signaling if a sender cell interacts with a receiver cell (Fig. 1B, bottom), but if both interacting cells are in the same signaling state (Fig. 1B, top and middle panels) much less signal is transduced.

Given that the Notch signaling system is involved in many developmental processes, it is important to determine how this *cis*-dependent send/receive signaling switch impacts pattern formation in developing tissues. A well-studied class of biological patterning systems is local self-activation with long-range inhibition [15]. Our model of Notch signaling-driven lateral inhibition patterning may be discussed in similar terms, with the mutual *cis* inhibition contributing to both the local and long-range effects. However, in this case the coupling required for “long-range” inhibition occurs via short-range nonlinear juxtracrine interaction between neighboring cells, instead of via linear diffusion of a signaling molecule across long distances [16]. Moreover, the mutual inactivation of Notch and DSL discussed above provides an improved source of intra-cellular self-activation [17] leading to the effects on pattern formation described here.

In order to understand the implications of the Notch-DSL signaling switch for developmental patterning, we analyzed mathematical models of two canonical developmental patterning processes: (1) morphogen gradient-driven boundary formation and (2) lateral inhibition. We compared models incorporating mutual inactivation in *cis* to alternative models lacking this interaction. The results show how mutual inactivation provides several key advantages for patterning circuits: it can allow sharp boundary formation without intracellular feedback, maintain it across a broad range of morphogen gradient slopes, and make patterning insensitive to correlated fluctuations (‘extrinsic noise’) in Notch and ligand expression. In lateral inhibition circuits, mutual inactivation speeds up patterning and relaxes parametric requirements on the regulatory interactions. Finally, it permits a surprisingly simple, and counter-intuitive, lateral inhibition circuit architecture, in which Notch activates its own expression, and no additional feedback or involvement of other components is required.

## Results

### Mutual inactivation, even in the absence of intracellular feedback, generates sharp boundaries

**Figure 2:**
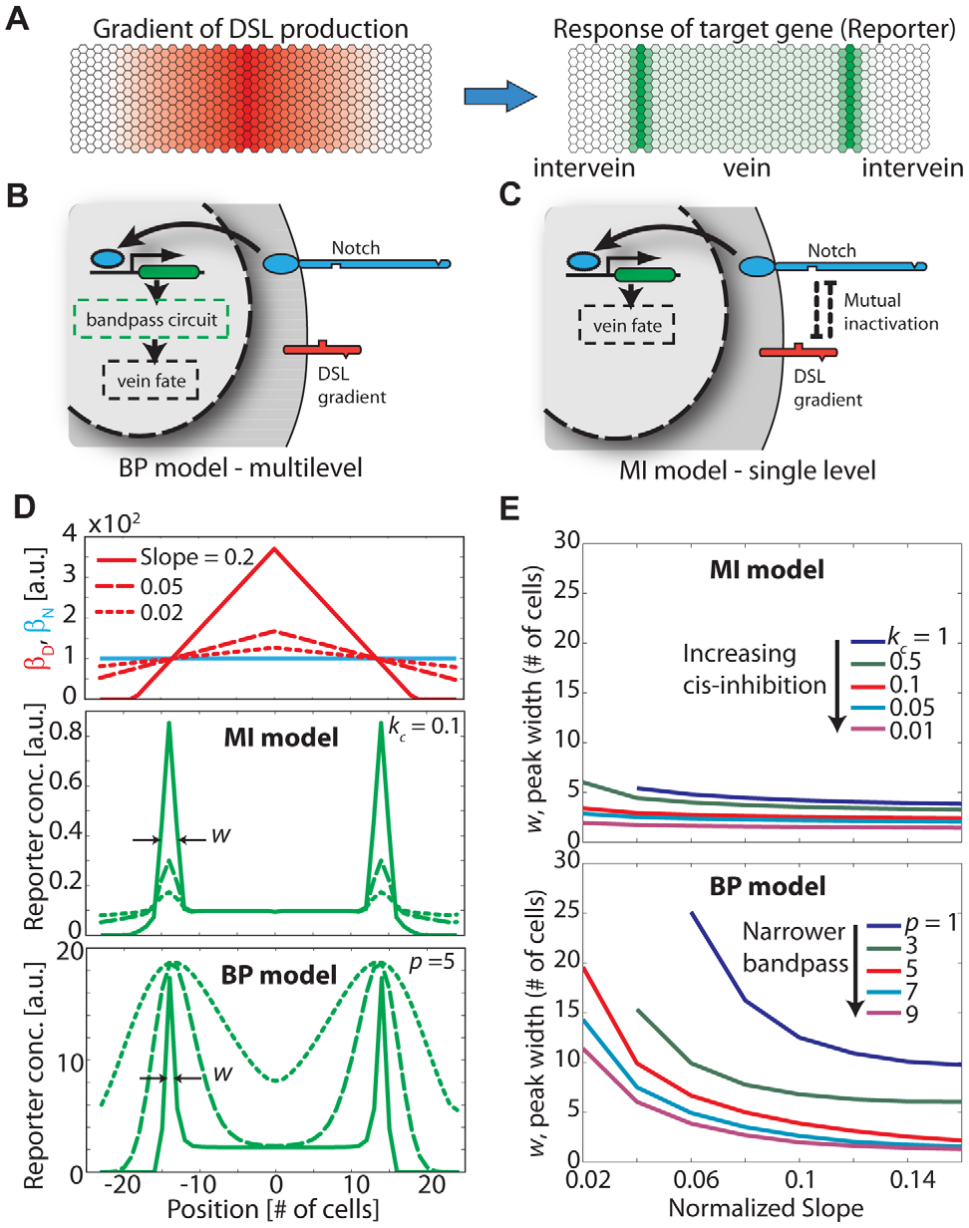
Mutual inactivation facilitates wing vein boundary formation. (A) Schematic of vein boundary formation. During vein formation a gradient in DSL production from the center of the vein (left, red) is converted into two sharply defined sidebands of Notch target expression (right, green). (B) Cartoon of the Bandpass regulatory mechanism, in which the boundary is determined by a transcription-level filter which determines the mapping from Notch activity to cell fate. Note that there is no feedback on the signaling system. (C) Cartoon of the Mutual Inactivation model regulatory mechanism, in which the level of Notch signaling directly determines the cell fate. Note again that there is no feedback on the signaling system. (D) Simulations of boundary formation. Top: DSL gradient profiles (three red curves) with varying slopes, chosen to generate side bands at a fixed position. Middle, bottom: Profiles of target reporter concentrations for the three slopes shown in the top panel for the MI model (middle) and the BP model (bottom). (E) Dependence of peak width on slope for the two models. In the MI model (top panel), peak width, *w*, remains small over a range of gradient slopes and strengths of the mutual inactivation interaction, 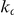. Here, smaller 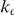 corresponds to stronger *cis*-inhibition (See Eqns. 1–2). In the BP model (bottom panel) peak width depends on the gradient slope as well as on the bandpass steepness parameter, 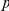. Here, higher 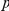 corresponds to a steeper bandpass (see Eqn. 6 and Fig. S1). Note that for the BP model, DSL production profiles were shifted to lower levels (see Table S1) but maintained the same slopes compared to the profiles shown in (B, top). This made sure that the bandpass is in a functional regime in which Notch signaling varies linearly with position (e.g. as in Fig. S1B). See Table S1 for parameter values.

Wing vein formation in the developing fly is a classic model system for studying the generation of sharp boundaries. In the *Drosophila* wing, there are four longitudinal veins that include several rows of cells that are more compact and have darker pigmentation than intervein cells. The position of the wing veins in the wing imaginal disk is initiated by EGF signaling during the early stages of larva development [18]. The final form (position and width) of the wing veins is refined by several subsequent processes. Notch signaling has been shown to specifically control the sharpening of the boundary between pro-vein (the region competent to produce vein fates) and intervein regions in the wing disc [1], [2]. In this system, the Delta production rate is controlled by a gradient of *veinless* expression diminishing outward from the center of the pro-vein region (Fig. 2A, left). Notch signaling is observed in two sharply defined side-bands, which restrict further vein development to the region between them (Fig. 2A, right).

We analyzed two simplified models of boundary formation, with or without mutual inactivation (Figs. 2BC, Eqns. 1–6, and Supporting Information Text S1.2). In both models, we assume constant Notch production (at a rate denoted 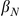) throughout the field of cells (blue line in Fig. 2D, top). We also assume that a linear gradient from the center of the vein, 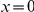, controls the rate of ligand production, denoted 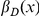 (red lines in Fig. 2D, top). Alternative models with other gradient shapes lead to the same results shown below.

In the mutual inactivation (MI) model (Fig. 2C), mutually exclusive signaling states generate sharp side-bands (as observed experimentally) where ‘sender’ cells contact ‘receiver’ cells near the crossing of the Notch and DSL production rate profiles. This model does not consider any feedback of Notch signaling on either DSL or Notch itself in the same cell, and thus lateral inhibition does not arise in this case (in contrast with the lateral inhibition models below).

Alternatively, in the ‘bandpass’ (BP) model a similar Notch activity profile can be generated in the absence of mutual inactivation, but this requires a bandpass filter of Notch activity level which we represent phenomenologically as the product of increasing and decreasing Hill functions (Figs. 2B, S1A). Such a bandpass filter represents the effective action of diverse regulatory processes downstream of Notch signaling, which could exist in different signaling architecture alternatives to the MI mechanism. We note here that while transcriptional feedbacks on Notch and DSL have been described in vein formation [1], [2], we do not explicitly consider them in these models in order to focus on the main effects of the mutual inactivation process. Our qualitative conclusions are insensitive to their inclusion. The equations representing these models are derived in the Supporting Information Text S1.2 and summarized in Eqns. 1–6.

### Mutual inactivation makes boundary sharpness insensitive to morphogen gradient slope

The slope of the morphogen gradient is expected to vary in natural systems from fluctuations and/or genetic variability, and thus may be an important factor in determining boundary features. To investigate the effect of such variability on boundary formation, we systematically analyzed the responses of the two models to different morphogen gradient slopes. For both models, we maintained the position of the threshold at a constant distance from the center of the vein (Fig. 2D, top).

In the MI model, the width of the signaling bands remained nearly constant across a wide range of morphogen gradient slopes (Fig. 2D, middle). This resulted from the sharp switch from a sending to a receiving state at the 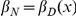 intersection. In contrast, the amplitude of the signaling bands changed systematically with the magnitude of the slope. This can be understood by considering how much free Notch and free DSL is available at the sender-receiver interface. The concentration of free DSL or Notch in the sending or receiving cell, respectively, is approximately proportional to the difference in Notch and DSL production rates, which in turn is proportional to the slope of the gradient.

In contrast, the BP model shows substantial broadening of the bands at lower values of the gradient slope (Fig. 2D, bottom). Unlike in the MI model, here Notch signaling occurs throughout the field of cells and is simply filtered by the downstream band-pass. As a result, the width of the Notch signaling bands is approximately proportional to the width of the bandpass divided by the slope of the Notch signaling profile (Fig. S1B).

The key parameters controlling the reporter expression profiles are the strength of the *cis*-interaction, 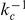 for the MI model (decreasing 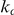 leads to increasing *cis*-interaction strength), and cooperativity, 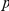 for the BP model. Interestingly, the BP model supports a sharp boundary only for sufficiently large 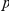 and sufficiently high slopes (Fig. 2E, bottom). In contrast, with the MI model, band sharpness is preserved across a broad range of 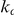 values and morphogen slopes (Fig. 2E, top). Thus, mutual inactivation enables a more robust patterning mechanism.

### Wing vein mutant behavior is explained by the MI model

**Figure 3:**
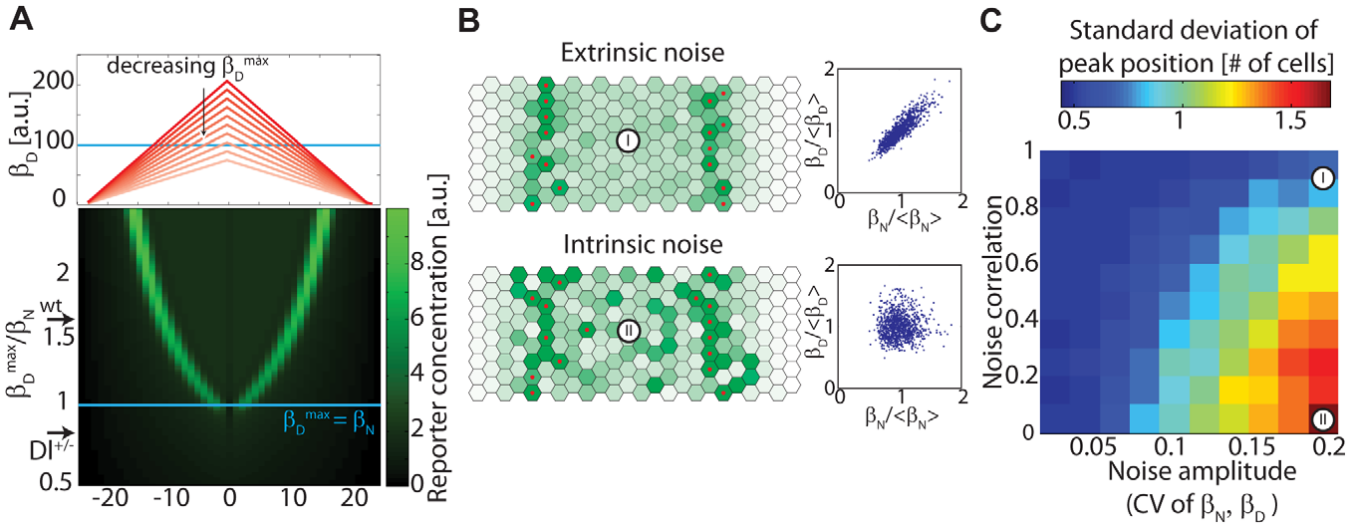
Boundary width is robust to correlated noise in Notch and Delta. (A) Notch reporter profiles (green heat map, bottom panel) for varying maximal production rates of DSL, 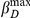 (red curves in top panel) and a fixed production rate of Notch, 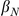 (blueline). Spatially-uniform reduction in 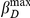 levels (*y*-axis, lower panel) results in restriction of the vein to a progressively narrower region (lower panel). However, when the DSL production rate is lowered to the extreme when 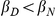 everywhere, all cells are in receiver states, and vein boundaries are no longer restricted by Notch signaling (see discussion in text). This is the expected behavior in the Delta^+/−^ heterozygous mutant when the DSL production rate is half that of the wild-type (arrows), if in the wild type 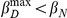. (B) The Notch reporter profile is sensitive to intrinsic (uncorrelated) noise but robust to extrinsic (correlated) noise in Notch and DSL production rates. Simulations of boundary formation with static multiplicative production rate noise of similar magnitude but different degrees of correlation (blue scatter plots) show that the pattern is less sensitive to extrinsic noise (top) than intrinsic noise (bottom). (C) The effect of noise amplitude and degree of correlation on Notch reporter peak positions. Standard deviation in peak position (color bar) at each row (red dots in B) is calculated from 300 simulations of 8×24 cell arrays (such as those in B) for different noise attributes. The noise parameters used in B are marked (white circles). See Supporting Information Text S1.5 and Table S1 for parameter values and description of noise generation.

A striking aspect of the *Drosophila* wing vein system is observed in the heterozygous mutants of Notch and Delta (e.g. single copies of the Notch and Delta genes). While heterozygous mutants of Notch (Notch^+/−^) or Delta (Delta^+/−^) alone exhibit mutant phenotypes (causing thicker veins), the Notch^+/−^ Delta^+/−^ double mutant restores the wild-type phenotype [19], [20], [21]. More generally, several mutant phenotypes seem to depend on the ratio between the copy numbers of the Notch and DSL genes [19]. This ratiometric dependence of the vein width cannot be derived from the several known feedbacks operating in the *Drosophila* wing vein, but emerges automatically from the MI model. This is because the position of the Notch signaling band occurs where Notch and DSL production rates are equal. This position remains unchanged when both rates are multiplied by the same factor. By the same reasoning, the vein width (distance between side bands) increases with increasing ratios between the effective copy numbers of DSL and Notch, as shown in Figs. 3A, S2.

Interestingly, however, this picture breaks down when the maximum DSL production rate, 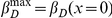, becomes smaller than the Notch production rate, 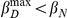. What phenotype would we expect in this case? Here, since all cells are essentially ‘receivers’ we expect negligible levels of Notch signaling, leading to a phenotype of an unsharpened, diffusely-defined vein, that defaults to the pre-patterned vein-competent region. Indeed, the Delta^+/−^ phenotype exhibits broad veins with diffuse boundaries, similar to Delta null mutant clones [19]. This result makes a quantitative prediction: the maximal DSL (Delta ligand in the case of the wing vein) production rate should be less than twice the constitutive Notch production rate in this system.

### Mutual inactivation-based boundary formation is sensitive to intrinsic noise but robust to extrinsic noise

In the fly larva, the width of the vein remains quite constant over length-scales of many cells. This occurs despite the possibility of substantial fluctuations, or ‘noise’, in the expression of Notch, Delta, and other components [22]. In order to understand how gene expression noise affects the MI wing vein model, we considered the response of the system between two limiting cases [23]. At one extreme, noise can be completely ‘intrinsic’, meaning that Notch and DSL production rates fluctuate in an uncorrelated manner. At the opposite extreme, ‘extrinsic’ noise could dominate, generating correlated fluctuations in Notch and DSL production. As shown in Fig. 3B, intrinsic noise causes the width of the vein to become irregular (Fig. 3B, bottom), while extrinsic noise of the same magnitude has significantly less effect on width (Fig. 3B, top). To show the generality of this effect, we performed simulations of boundary formation patterning for a range of different noise amplitudes and correlations (Fig. 3C). These simulations show that the standard deviation of peak position (which is a measure of pattern robustness) decreases as the noise becomes more extrinsic.

This behavior emerges from the ratiometric sensitivity of the MI model to the levels of Notch and DSL. In the MI model, the signaling state of a cell (sending or receiving) is determined by the ratio of Notch to DSL – in ‘sender’ cells this ratio is smaller than one, and in ‘receivers’ it is greater than one. As the vein edge is defined by Notch signaling, it is restricted to the area where sender cells are in direct contact with receiver cells, at which Notch and DSL production rates are comparable (Fig. 2D). Extrinsic noise tends to maintain constant relative expression of Notch and DSL. Therefore, it does not disturb the segregation of cells into senders and receivers, and preserves the band of Notch signaling activity. This effect is maintained across a broad range of noise amplitudes and correlation levels.

### Mutual inactivation speeds lateral inhibition patterning

**Figure 4:**
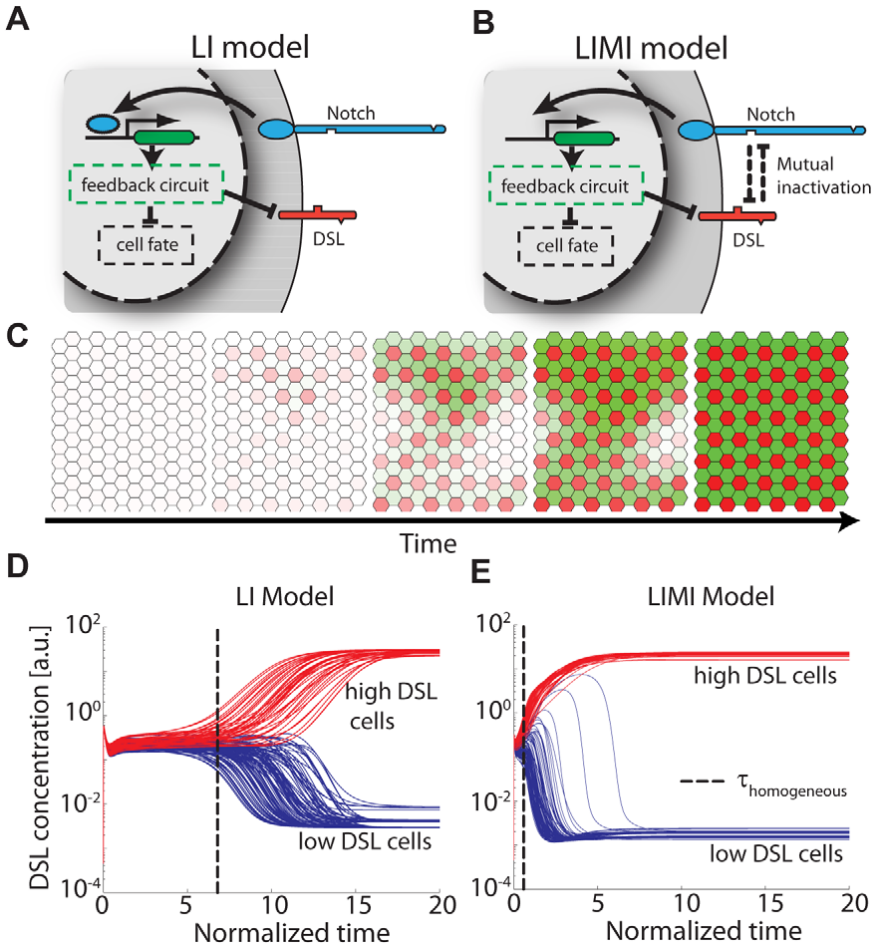
Mutual inactivation facilitates lateral inhibition patterning with faster dynamics. Comparison between (A) standard lateral inhibition model (LI) and (B) lateral inhibition with mutual inactivation (LIMI). (C) A typical simulation of lateral inhibition dynamics showing pattern generation from an initially homogenous steady state (HSS). (D–E) Simulations reveal that the LIMI model (E) patterns faster than the LI model (D). Red and blue curves show the dynamics of DSL levels in cells with high and low final DSL levels, respectively. Vertical dashed lines indicate the ‘homogeneous time’ defined as the time it takes the coefficient of variation to increase above 50% of its final value (see Fig. S3). These simulations were performed with the parameters indicated by the black dots in Fig. 5AB. Similar behavior is observed over most of the parameter space (see Fig. S3).

Lateral inhibition models have been used to describe the formation of checkerboard-like patterns in which high DSL cells are surrounded by low DSL neighbors. This type of structure occurs in bristle patterning in *Drosophila* [3]and hair cell patterning in the vertebrate inner ear [24]. Standard lateral inhibition (LI) models assume that neighboring cells inhibit each other's differentiation through Notch signaling, which indirectly down-regulates DSL expression to form an intercellular positive feedback loop (Fig. 4A, Supporting Information Text S1.3). Under the right conditions, this feedback loop can amplify small initial differences between cells and generate patterns in which neighboring cells exhibit alternating expression levels. A lateral inhibition model of this type was analyzed previously [16], [25].

**Figure 5:**
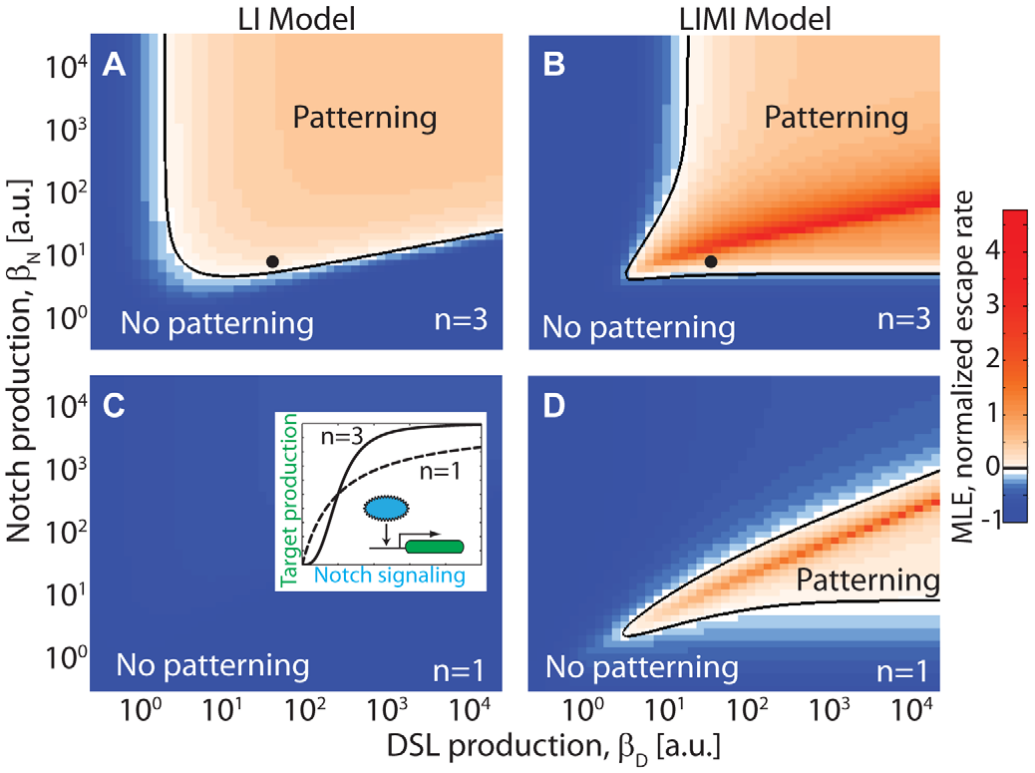
Mutual inactivation circumvents requirement for cooperative feedback. Escape rates from the HSS (indicated by Maximum Lyapunov Exponents, or MLE) as a function of 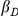 and 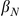. MLE values were calculated using linear stability analysis (Supporting Information Text S1.4) for the LI (A,C) and LIMI (B,D) models. Positive MLE values (white/pink regions) support patterning, while negative MLE values (blue regions) do not. The dependence on feedback loop cooperativity (inset) can be seen by comparing (A,B) to (C,D). Black dots in A and B correspond to the parameters used to simulate Fig. 4DE.

How does mutual inactivation affect the lateral inhibition patterning process? To address this question we systematically compared the standard LI model (Fig. 4A) to a lateral inhibition with mutual inactivation (LIMI) model (Fig. 4B, equations are summarized in Eqns. 10–12, and derived in Supporting Information Text S1.3). Because the MI interaction constitutes an additional, rapid intracellular feedback, we intuitively expected an effect on both the patterning speed and accessibility. To test this hypothesis, we performed dynamical simulations to determine patterning speed, and linear stability analysis about the system's homogeneous steady state (HSS) to determine pattern accessibility. The HSS is defined as the steady state in which all cells have identical concentrations of signaling system components [16], [26].

Using dynamical simulations, we first compared how rapidly the LI and LIMI models are able to reach the patterned state from an initially non-patterned state. Fig. 4CDE shows the dynamics of DSL concentration in single cells for both models with one set of parameters (black dot in Fig. 5). The LI model initially spends a considerable time in a nearly homogeneous state (left of the dashed line in Fig. 4D) before DSL concentrations diverge (red and blue curves, right of the dashed line). In contrast, in the LIMI model, DSL concentrations diverge much earlier (Fig. 4E). The LIMI process approaches the final patterned state more rapidly than the LI process, largely due to the difference in the rate of deviation from homogeneity. A similar difference in the patterning speed is observed over a large region in parameter space as shown in Fig. S3CDEF.

Why are the dynamics accelerated in the LIMI model? A key difference in the LIMI model is the inactivation terms, which are equivalent to effective degradation terms (e.g. 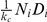). Because protein degradation is assumed to be the slowest timescale in the system, increasing the degradation rate speeds up the overall response time. In principle such acceleration could be achieved in the LI model as well, just by increasing the magnitude of the constitutive degradation terms. Note, however, that in the LIMI model the additional degradation only occurs when both Notch and DSL are simultaneously present on the same cell. This causes an acceleration specifically during patterning, while avoiding unnecessary protein turnover that would result from increased constitutive degradation.

### Mutual inactivation allows lateral inhibition without cooperative interactions

The potential for lateral inhibition pattern formation in a given system is strongly controlled by its dynamical behavior near the HSS. For some parameter sets, the HSS is stable and no patterning occurs. For other parameter sets, the HSS is unstable. In this case, although components' concentrations may initially approach their HSS values, in the presence of even arbitrarily small heterogeneous fluctuations they must subsequently diverge, generating the patterned state (Fig. 4C).

We next set out to systematically compare the patterning ability of the LI and LIMI models. We performed linear stability analysis of the HSS [16], [26] across a broad range of parameter values, and determined the subset of parameter sets for which the system's HSS is unstable to perturbations (Fig. 5A–D). Formally, this is done by calculating the maximal escape rate from the non-patterned HSS (Supplementary Information S4). If this rate, termed the Maximal Lyapunov Exponent (MLE), is positive, the HSS becomes unstable and patterning occurs.

In Fig. 5A–D we plot the MLE as a function of the production rates 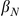 and 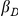 for two different effective cooperativities, for both the LI and LIMI models. At high cooperativity (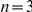), both models show a large region of parameter space in which the system patterns (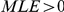) (Fig. 5AB), although quantitatively the LIMI MLE is generally greater than the LI MLE. In contrast, when 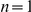, only the LIMI model supports patterning anywhere in the parameter space (Fig. 5CD). Thus, the mutual inactivation model circumvents the requirement for cooperative regulatory feedback in the standard lateral inhibition model. The qualitative behavior of Fig. 5A–D is maintained as long as the *cis* interaction is strong enough (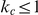).

### Mutual inactivation permits lateral inhibition patterning with only a single level of transcriptional feedback

**Figure 6:**
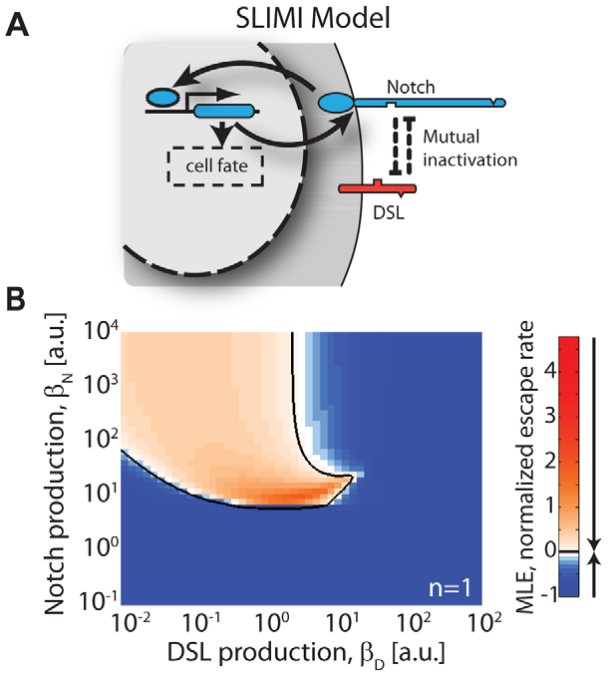
Simplified lateral inhibition with mutual inactivation (SLIMI). (A) Schematic of a simplified lateral inhibition circuit architecture. Here, Notch signaling activates expression of the Notch gene. Notch activation thus leads to higher Notch levels which, in turn, lead to lower levels of free DSL due to the mutual inactivation interaction between Notch and DSL proteins in *cis* (dashed arrows). (B) Calculation of the MLE for the SLIMI model. The SLIMI model can support patterning without cooperative feedback over a large region of parameter space. Color scale is the same as in Fig. 5A–D. Equations and parameters are described in the Supporting Information Text S1.3 and Table S1, respectively.

Mutual inactivation can have a more dramatic effect on patterning: Besides improving the performance of standard patterning circuits, it can enable an altogether different, and simpler, lateral inhibition circuit architecture. The essential requirement for lateral inhibition is that increased Notch activity in one cell reduces its ability to signal to its neighbors. In the presence of mutual inactivation, one way to achieve this is for Notch activity to directly up-regulate Notch expression (Fig. 6A). Increased levels of Notch result in more rapid removal of DSL through the mutual inactivation interaction, effectively down-regulating it. Thus, a circuit in which Notch activates its own expression implements lateral inhibition with only a single level of transcriptional feedback, i.e. instead of Notch activating a repressor of DSL, there is direct downregulation of DSL through the mutual inactivation interaction. This type of autoregulation has been observed in some cases, such as the *C. elegans* AC/VU fate determination system [27]. We term this circuit architecture ‘Simplest Lateral Inhibition with Mutual Inactivation’ (SLIMI). Linear stability analysis of this SLIMI circuit (Fig. 6B) shows that patterning can occur across a broad range of parameter values. Moreover, as with the LIMI model, SLIMI does not require explicit cooperativity for patterning. Thus, lateral inhibition can be achieved with a startlingly simple circuit architecture.

## Discussion

Even as the molecular components of patterning circuits become increasingly known, the ways in which these components interact dynamically to generate patterns often remains unclear. We and others recently reported evidence for a strong mutual inactivation interaction that occurs between Notch and DSL in the same cell [11], [12]. Mutual inactivation between Notch and DSL is a relatively simple biochemical mechanism that generates an ultrasensitive, cell-autonomous, switch between “sending” and “receiving” states (Fig. 1). Other mechanisms such as cooperative binding of transcription factors and regulatory feedbacks can also generate switch-like responses, but they require a more complex regulatory setup (e.g. multiple binding sites, DNA looping, or more elaborate gene circuits) and are rarely observed to have effective cooperativity higher than 3 or 4 [28], [29], [30], [31]. More generally, sequestration interactions are emerging as a widespread mechanism for sharp switching in diverse biological systems [32], [33], [34], [35], [36], [37], [38].

These and other experimental observations necessitate a revised analysis of patterning circuit mechanisms [10], [11], [12]. As an initial step, we have used mathematical modeling to analyze two canonical Notch-dependent patterning processes: the formation of sharp boundaries in the *Drosophila* wing vein [1], [2] and the formation of alternating patterns of differentiation, such as that found in *Drosophila* SOP patterning [3], [39]. The results described here show that mutual inactivation facilitates these patterning processes, and permits simpler regulatory architectures.

### Boundary formation

In the wing vein boundary, graded expression of Delta is converted to two sharply defined ‘side bands’ of Notch activity [1], [2]. The mutual inactivation mechanism achieves this conversion without requiring additional circuit components. Furthermore, unlike a broad class of alternative models based on transcriptional cooperativity (e.g. the BP model), the MI model can generate sharp boundaries over a wide range of gradient profiles and biochemical parameters (Fig. 2DE).

The MI model has a unique property that can experimentally distinguish it from other models: The pattern of expression of Notch target genes depends on the *relative* expression levels of Notch and DSL rather than on their absolute concentrations (Figs. 3A, S2). This property can explain the ratiometric behavior observed in Notch and Delta heterozygous mutants [19], [20] (Fig. S2). Interestingly, when DSL expression in our model is reduced below Notch expression level everywhere, very little signaling occurs (below the blue line in Fig. 3A). In this condition Notch signaling is no longer expected to restrict vein width, resulting in a broader vein with diffuse boundaries [2]. This leads to the following experimental prediction: by reducing Delta production *continuously*, the width of the veins should first decrease as the crossing points between Notch and Delta production rates move toward the center of the vein. However, this thinning should be followed by an abrupt switch to the unrestricted (wider) vein regime once 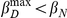 (Fig. 3A).

The same ratiometric behavior also underlies the dependence of the pattern on noise (Fig. 3BC): while the width of the boundary is sensitive to intrinsic noise (uncorrelated between Notch and DSL) it is robust to extrinsic noise (correlated between Notch and DSL). Experimental measurements of the correlations between Notch and Delta expression in wing discs (or other systems) would help to determine which noise regime is most relevant *in vivo*.

We note that transcriptional feedback of Notch signaling on Notch and Delta expression has been shown to occur in the *Drosophila* wing vein boundary [1], [2]. Here we have omitted these feedbacks in order to focus specifically on the effects of mutual inactivation. However, it is important to note that these feedbacks are not sufficient to explain the experimentally observed ratiometric behavior (Fig. S10 in ref [11]). Experimental disruption of these feedbacks could help to determine what role they play in patterning, e.g. whether they function to control the pattern itself, to increase its amplitude, or to provide some other functionality in normal development.

### Lateral inhibition

Mutual inactivation facilitates lateral inhibition patterning in several ways. First, mutual inactivation accelerates patterning dynamics compared to an equivalent model without it (Fig. 4DE). The LIMI model accelerates dynamics by increasing protein turnover, but does so selectively only when both proteins are present on the same cell. Thus, once patterning is complete, there is no additional protein turnover cost. Notch has been shown to exhibit relatively fast response times in some systems, and the lifetime of the cleaved intracellular domain of Notch is short and highly regulated [40], suggesting that the acceleration provided by mutual inactivation could be important in development. Furthermore, recent work has attributed minimization of errors in patterns of the sensory organ precursors to faster dynamics due to *cis*-inhibition [21].

A second advantage is that mutual inactivation removes the requirement that would otherwise exist for an explicitly cooperative step in the lateral inhibition feedback loop (Fig. 5). This requirement on the LI model was previously proven analytically both for a 1D chain [16] and a 2D [11] hexagonal lattice. In fact, mutual inactivation plays a dual role here: in addition to providing the non-linearity required for the amplification of small differences between neighboring cells, it also introduces an additional intracellular feedback reinforcing the intercellular feedback loop. When Notch signaling down-regulates DSL, this also reduces the rate of Notch inactivation, effectively freeing additional Notch receptors and leading to an additional increase in Notch signaling.

Finally, mutual inactivation allows a new, alternative circuit architecture for lateral inhibition: Instead of transcriptionally down-regulating DSL, Notch can up-regulate its own expression (Fig. 6A). This architecture is sufficient for lateral inhibition patterning across a broad range of parameters (Fig. 6B). This alternative architecture is intriguing because in some natural lateral inhibition circuits the regulatory pathway for Notch-dependent down-regulation of DSL remains unclear [41], [42] (we note that in other systems downregulation of DSL by Notch has been observed). At the same time, Notch up-regulation by Notch signaling has been shown in several lateral inhibition patterning examples, such as the AC/VU system in *C. elegans* [27]. This mechanism may provide the main feedback in lateral inhibition circuits, or may work in combination with the classical lateral inhibition feedback mechanisms on DSL (LIMI model). It will be interesting to determine to what extent this mechanism participates in various lateral inhibition systems.

In general, mutual inactivation of Notch and DSL in *cis* may be conceived as a direct, rapid, and sharp replacement for an additional level of intracellular feedback that would otherwise have been required to drive neighboring cells to distinct fates in a fine-grained spatial pattern. In this sense we may say that an intrinsic difference-promoting logic is encoded in the signaling system itself by the mutual inactivation phenomenon. Because of this, regulatory circuit architecture that achieves fine-grained patterns without MI can be made less complicated (i.e. with fewer regulatory levels) by including MI. Both examples analyzed here demonstrate this feature.

Together the results above provide a theoretical framework as well as testable hypotheses for the role of mutual inactivation between Notch and DSL in the generation of fine-grained developmental patterns. In the future, this analysis can be expanded to include additional circuit details such as further regulatory feedbacks, multiple Notch ligands and receptors, and modifiers of Notch signaling, and extended to additional Notch-dependent patterning systems.

## Materials and Methods

In summary, our model consists of three protein components – Notch (*N*), DSL (*D*), and a Reporter (*R*) – with two basic interactions: between Notch and DSL on different cells (in *trans*) to stimulate reporter production in the Notch-bearing cell, and between Notch and DSL on the same cell (in *cis*) to unproductively inactivate both molecules. These interactions are parametrized by the following quantities:


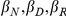
: production rates of Notch, DSL, and Reporter target gene, respectively.


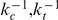
: the strengths of the *cis*- and *trans*-interactions, respectively.


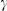
: degradation rate of Notch and DSL, assumed to be equal for simplicity (no loss of generality for the steady state solutions, see Supplementary).


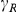
: degradation rate of 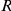.


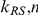
: affinity and Hill coefficient, respectively, of Reporter induction by Notch signaling.


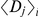
: average concentration of DSL in all cells, indexed by 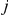, that are neighboring cell 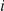. Similarly, 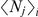 denotes the average concentration of Notch in all neighbors of the 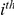 cell.

The description of the model in this section omits the dynamics of the *cis* and *trans* intermediate complexes, the Notch intracellular signaling domain, and the mRNAs corresponding to each protein. Formally, this is exact in the limit where these components' dynamics are rapid relative to that of the proteins. The former two of these conditions is reasonably expected to be valid. The Supporting Information presents the model in full detail, and contains a demonstration that including finite mRNA lifetimes does not modify our conclusions (Fig. S4). We also note that the model considered here is insensitive to the exact mechanism for *cis*-inhibition and whether the *cis* interaction occurs at the surface or not.

### MI model of boundary formation:



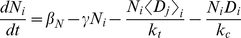
(1)

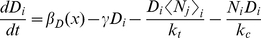
(2)

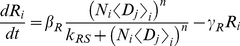
(3)


### Bandpass (BP) model for boundary formation:



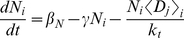
(4)

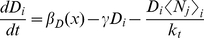
(5)

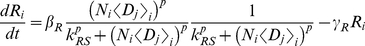
(6)


Compared to the MI model, these equations remove the *cis*-inhibition terms from the rates of change in Notch and DSL, and the production rate of the reporter 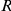 is now the product of two Hill functions, one decreasing and one increasing, with affinity 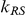 and cooperativity 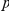.

### Lateral Inhibition (LI):



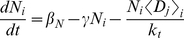
(7)

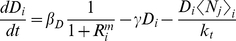
(8)

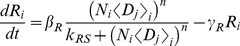
(9)


The parameters are defined consistently with the above. In these equations there is no *cis*-inhibition. The lateral inhibition is implemented by decreasing the production rate of DSL as a function of signaling Reporter levels, by the 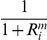 factor.

### Lateral Inhibition with Mutual Inactivation (LIMI):



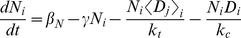
(10)

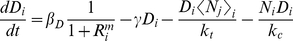
(11)

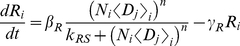
(12)


These differ from the LI model only by the inclusion of an additional *cis*-inhibition degradation term (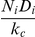) to the dynamics of both Notch and DSL.

### Simplest Lateral Inhibition by Mutual Inactivation (SLIMI):



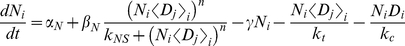
(13)

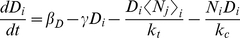
(14)


Because of the mutual *cis*-inhibition, upregulation of Notch expression in response to Notch signaling (represented as an increasing Hill function with strength 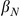, affinity 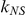, and cooperativity 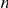) can implement lateral inhibition.

### Numerical computations

Dynamical simulations were performed using Matlab's ode15s solver (ver. 7.6.0, The Mathworks). Figs. 2DE were generated by solving Eqs. 1–3 for the MI model and Eqns. 4–6 in the BP model. Simulations were performed on a 12x48 hexagonal cell array assuming periodic boundary conditions. The DSL production profiles used were 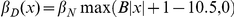 for the MI model and 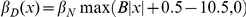 for the BP model, where 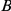 are the indicated slopes. Fig. 3A was generated using Eqns. 1–3 with DSL production rate profiles given by 
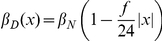
, where 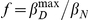 is as indicated in the figure. Figs. 3BC were generated using Eqns. 1–3 with multiplicative (static) noise terms for 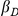 and 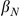. Generation of noise is described in Supporting Information Text S1.5. Figs. 4CDE were generated by solving Eqns. 7–12. These simulations were performed on a 12x12 hexagonal cell array assuming periodic boundary conditions. The MLE values in Fig. 5A–D were calculated by performing linear stability analysis on Eqns. 7–12 using previously described techniques ([16], Supporting Information Text S1.4). Parameters used throughout the analysis are provided in Table S1.

## Supporting Information

Figure S1Properties of the bandpass function in the BP model. (A) Bandpass profiles for different cooperativities 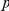. Reporter production rate is proportional to a bandpass function given by 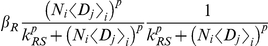 (first term in the right hand side of Eqn. 6). Here, the input, 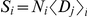, is the concentration of cleaved Notch intracellular domain. Increased 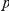 corresponds to narrower bandpass function. (B) Width of reporter peaks in the BP model (Fig. 2D, bottom panel) is proportional to width of bandpass function and inversely proportional to slope of gradient. A schematic showing the widths of the reporter peaks (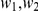) for a given bandpass width (on *y*-axis) and two gradient profiles (slope 1, slope 2).(TIF)

Figure S2Ratiometric dependence of vein width on Notch and DSL production. The distance between the two reporter peaks for the MI model (shown in Figs. 2D, 3A) as a function of the production rates 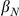 and 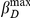. Vein width is maintained when the ratio between production rates is the same. This ratiometric dependence explains why the double heterozygous mutant (N^+/−^D^+/−^) exhibits similar veins to the wildtype (wt) while the single heterozygous mutants show mutant phenotypes (four white circles). Here, the D^+/−^ mutant falls in the ‘receiving only’ regime (below the blue line in Fig. 3A) where very little Notch signaling is produced across the field of cells. In this case, the vein is not restricted by Notch signaling leading to a broad vein with diffused boundaries. Parameters for the presented simulations are given in Table S1.(TIF)

Figure S3 Faster patterning dynamics in the LIMI model. (A,B) Determination of homogeneous time and total time for patterning. Time course of the coefficient of variation (CV) of DSL concentration (black solid line) is plotted for the data shown in Fig. 4DE (faded red and blue) corresponding to the LI (S3A and 4D) and LIMI (S3B and 4E) models. Homogeneous time, *τ_homogeneous_*, (dashed line) is defined as the time at which the CV is 50% of its final value. The total time, *τ_total_*, (dotted line) is calculated as the time it takes for the median high-DSL cell (faded red) to reach 95% of its final value. (C,D) Overall speed of patterning (defined as 1/*τ_total_*) in the LI model (C) is lower than in the LIMI (D) model over a large range of parameters. (E,F) An even larger difference is observed for the homogeneous speed of patterning (defined as 1/*τ_homogeneous_*) between the LI (E) and LIMI (F) models. This shows that onset of heterogeneity occurs much faster in the LIMI model and that this difference has a major contribution to the overall faster patterning dynamics.
(TIF)

Figure S4Effect of finite mRNA lifetimes. (A,B,C,D) The explicit inclusion of finite mRNA lifetimes in our MLE calculation does not affect the sign of the MLE, and correspondingly does not change our conclusion regarding the ability of the system to pattern. This is illustrated here for the (A,C) LI and (B,D) LIMI models with 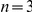, with (C,D) MLE plots for mRNA dynamics comparable to the first-order protein degradation rate and (A,B) extremely fast mRNA dynamics. (E,F) We also repeated our patterning speed analysis with slow mRNA dynamics and find that our qualitative conclusion that the LIMI model (F) accelerates patterning by more rapidly departing from the homogeneous state than the LI model (E) to be unchanged from the fast mRNA case, with only a quantitative change in the overall patterning time. As in Fig. 4DE, the traces of DSL concentrations over time are colored according to the eventual fate of the cell (red for high Delta, blue for low Delta). As in Fig. 3AB, the dashed black line demarcates the homogeneous and heterogeneous phases.(TIF)

Table S1 Details of parameters and references to equations used in figures.
(PDF)

Text S1 Details of derivations mentioned in text.
(PDF)
