# Supplementary figures and images for: Mutual Inactivation of Notch Receptors and Ligands Facilitates Developmental Patterning
Source: PLoS Comput Biol. 2011 Jun 9;7(6):e1002069. doi: 10.1371/journal.pcbi.1002069 (PMC3111533; doi:10.1371/journal.pcbi.1002069)

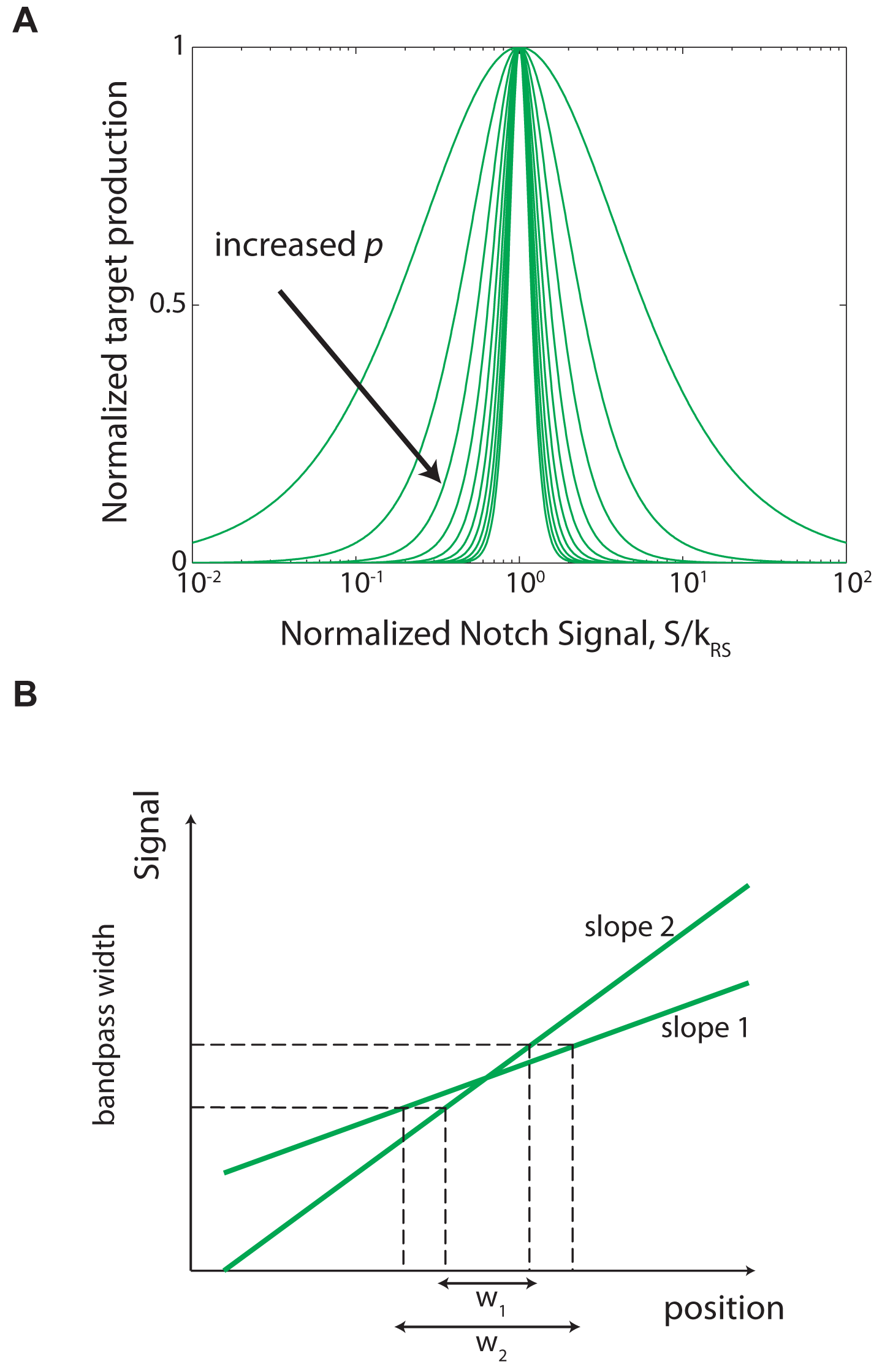

Supplement: Figure S1 — Properties of the bandpass function in the BP model. (A) Bandpass profiles for different cooperativities . Reporter production rate is proportional to a bandpass function given by (first term in the right hand side of Eqn. 6). Here, the input, , is the concentration of cleaved Notch intracellular domain. Increased corresponds to narrower bandpass function. (B) Width of reporter peaks in the BP model (Fig. 2D, bottom panel) is proportional to width of bandpass function and inversely proportional to slope of gradient. A schematic showing the widths of the reporter peaks () for a given bandpass width (on y-axis) and two gradient profiles (slope 1, slope 2). (TIF) [file pcbi.1002069.s001.tif]

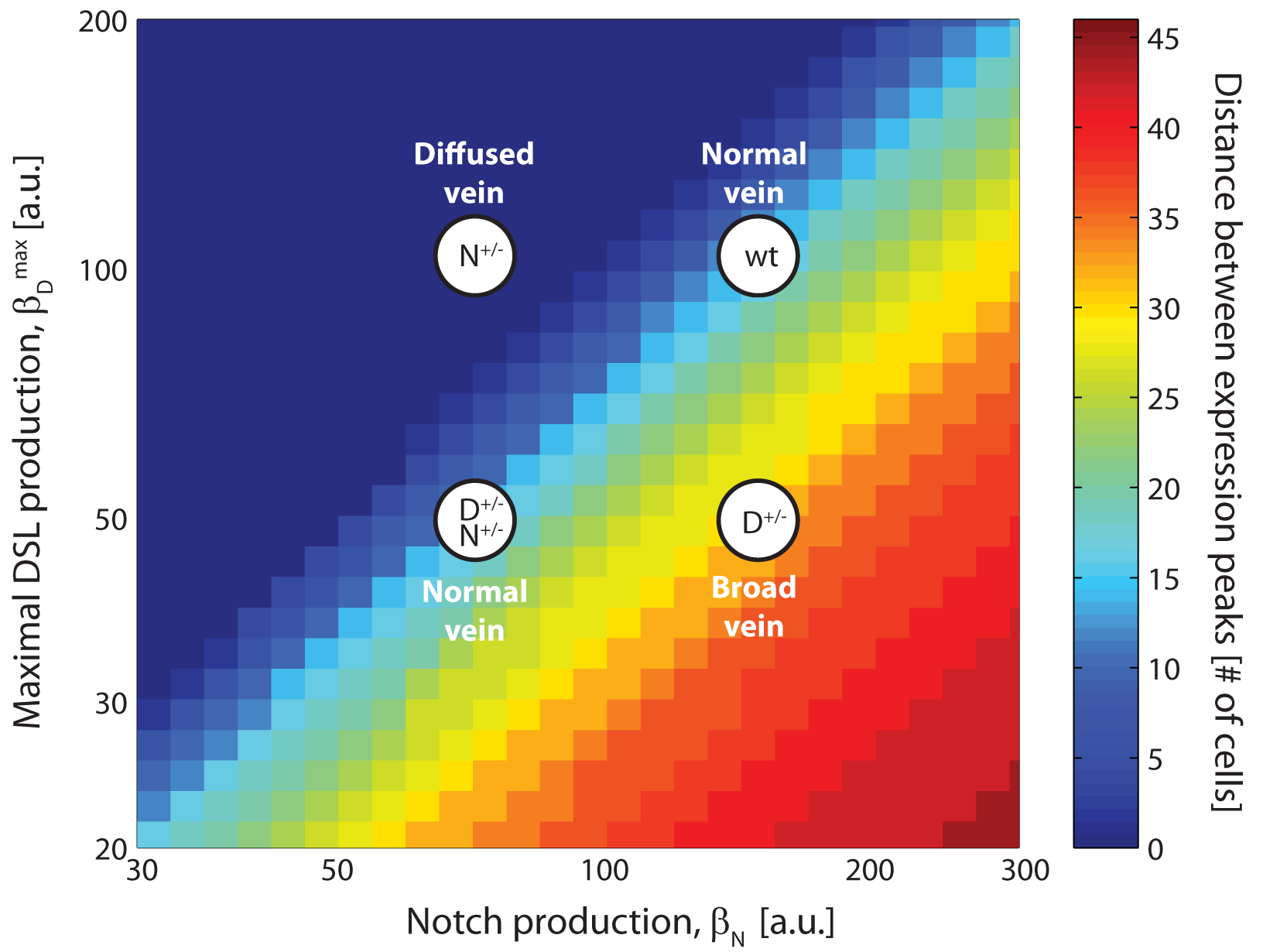

Supplement: Figure S2 — Ratiometric dependence of vein width on Notch and DSL production. The distance between the two reporter peaks for the MI model (shown in Figs. 2D, 3A) as a function of the production rates and . Vein width is maintained when the ratio between production rates is the same. This ratiometric dependence explains why the double heterozygous mutant (N+/−D+/−) exhibits similar veins to the wildtype (wt) while the single heterozygous mutants show mutant phenotypes (four white circles). Here, the D+/− mutant falls in the ‘receiving only’ regime (below the blue line in Fig. 3A) where very little Notch signaling is produced across the field of cells. In this case, the vein is not restricted by Notch signaling leading to a broad vein with diffused boundaries. Parameters for the presented simulations are given in Table S1. (TIF) [file pcbi.1002069.s002.tif]

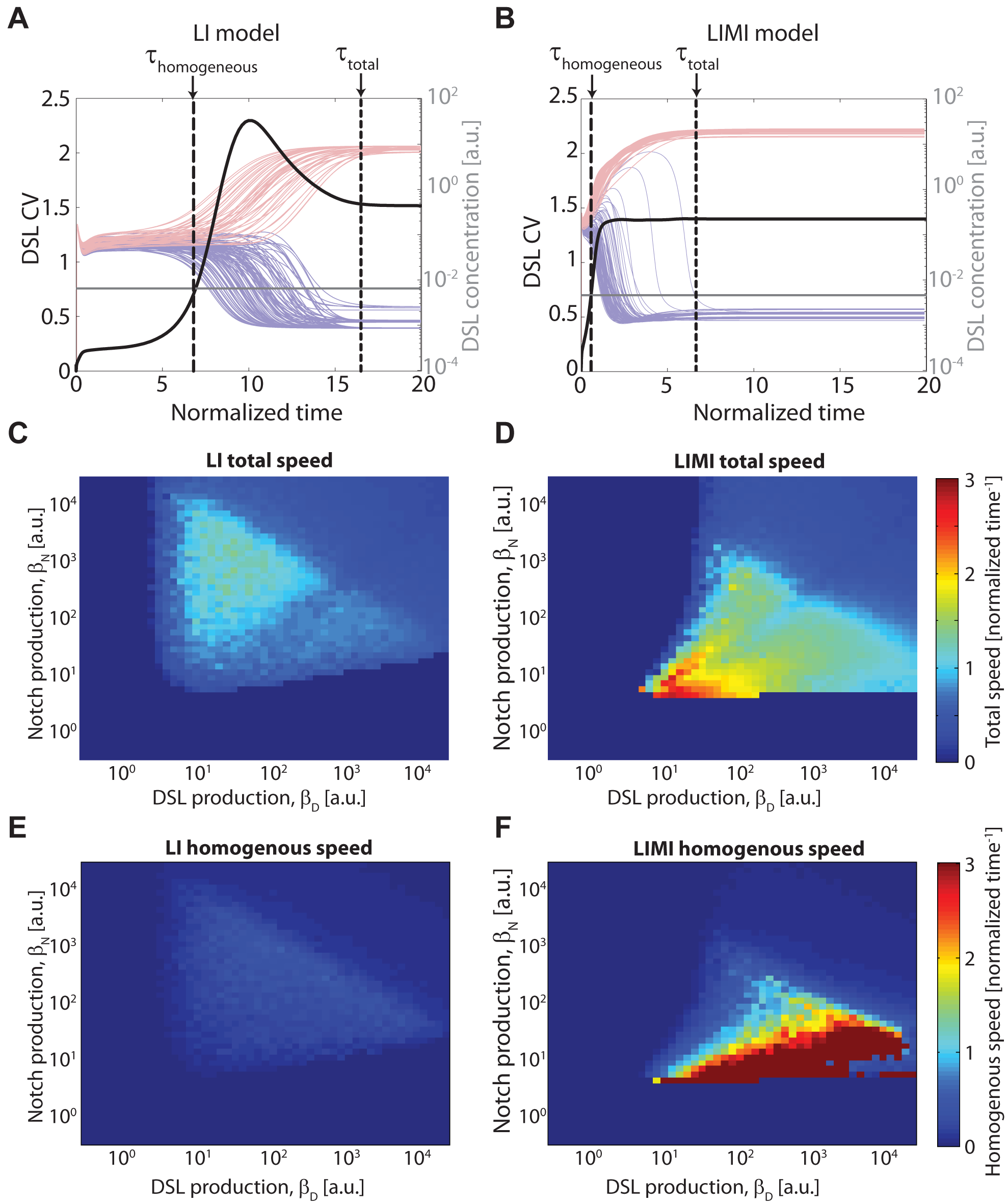

Supplement: Figure S3 — Faster patterning dynamics in the LIMI model. (A,B) Determination of homogeneous time and total time for patterning. Time course of the coefficient of variation (CV) of DSL concentration (black solid line) is plotted for the data shown in Fig. 4DE (faded red and blue) corresponding to the LI (S3A and 4D) and LIMI (S3B and 4E) models. Homogeneous time, τhomogeneous, (dashed line) is defined as the time at which the CV is 50% of its final value. The total time, τtotal, (dotted line) is calculated as the time it takes for the median high-DSL cell (faded red) to reach 95% of its final value. (C,D) Overall speed of patterning (defined as 1/τtotal) in the LI model (C) is lower than in the LIMI (D) model over a large range of parameters. (E,F) An even larger difference is observed for the homogeneous speed of patterning (defined as 1/τhomogeneous) between the LI (E) and LIMI (F) models. This shows that onset of heterogeneity occurs much faster in the LIMI model and that this difference has a major contribution to the overall faster patterning dynamics. (TIF) [file pcbi.1002069.s003.tif]

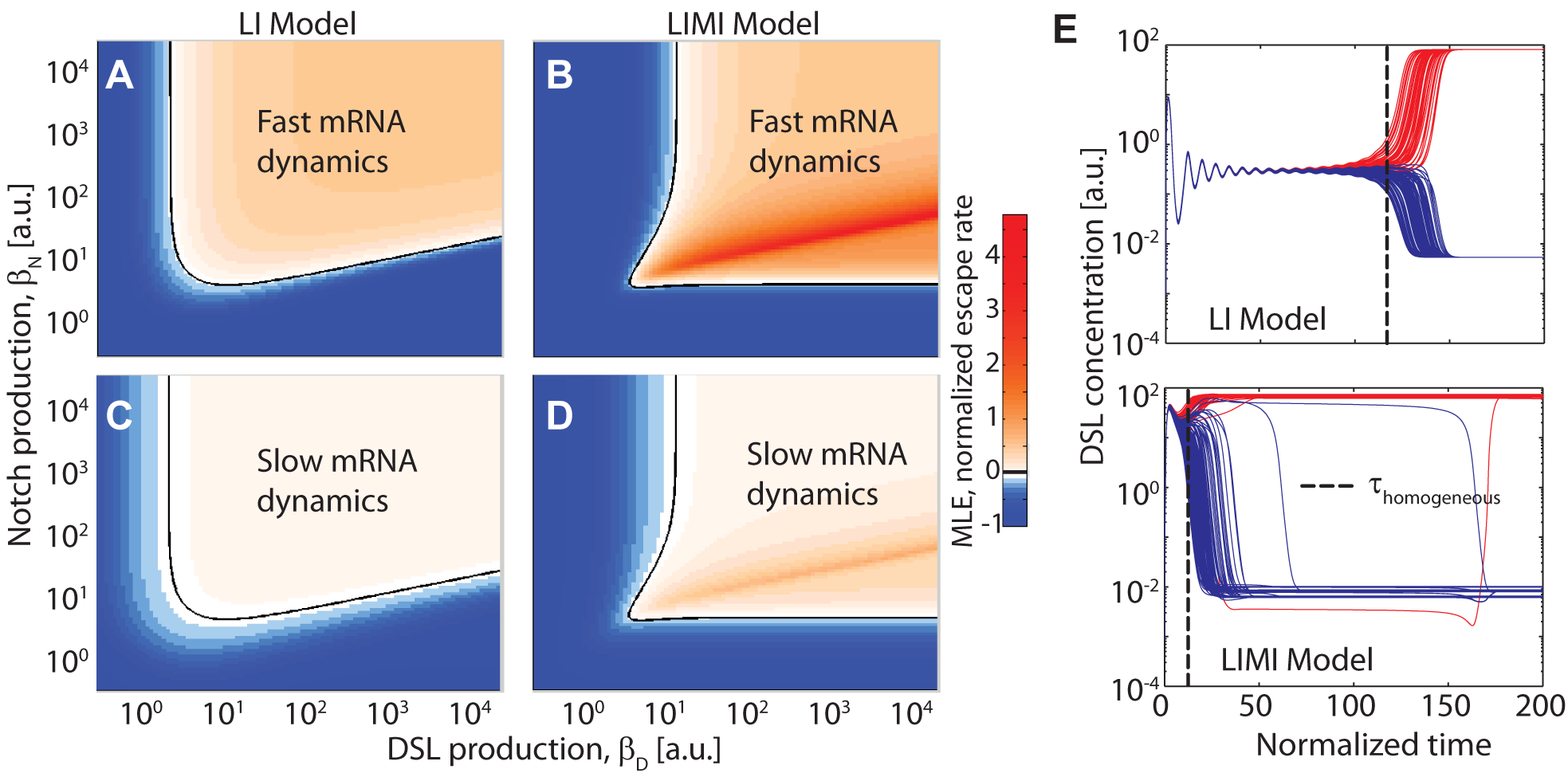

Supplement: Figure S4 — Effect of finite mRNA lifetimes. (A,B,C,D) The explicit inclusion of finite mRNA lifetimes in our MLE calculation does not affect the sign of the MLE, and correspondingly does not change our conclusion regarding the ability of the system to pattern. This is illustrated here for the (A,C) LI and (B,D) LIMI models with , with (C,D) MLE plots for mRNA dynamics comparable to the first-order protein degradation rate and (A,B) extremely fast mRNA dynamics. (E,F) We also repeated our patterning speed analysis with slow mRNA dynamics and find that our qualitative conclusion that the LIMI model (F) accelerates patterning by more rapidly departing from the homogeneous state than the LI model (E) to be unchanged from the fast mRNA case, with only a quantitative change in the overall patterning time. As in Fig. 4DE, the traces of DSL concentrations over time are colored according to the eventual fate of the cell (red for high Delta, blue for low Delta). As in Fig. 3AB, the dashed black line demarcates the homogeneous and heterogeneous phases. (TIF) [file pcbi.1002069.s004.tif]
