# Supplementary material for: Mutual Inactivation of Notch Receptors and Ligands Facilitates Developmental Patterning
Source: PLoS Comput Biol. 2011 Jun 9;7(6):e1002069. doi: 10.1371/journal.pcbi.1002069 (PMC3111533; doi:10.1371/journal.pcbi.1002069)
Supplement: Table S1 — Details of parameters and references to equations used in figures. (PDF) [file pcbi.1002069.s005.pdf]

# Supporting Table

## Table S1: Parameter values

| Figure | Equations | Parameters | Initial conditions and comments |
|---|---|---|---|
| 1A | S1.1.2a,b | $\gamma = 0.1$, $\gamma_S = 1$, $k_c = 1$, $k_t = 10$ , $\beta_N = 20$, $\beta_D = 0.02$-2000 | |
| 2D, middle | 1–3 | $\gamma = 0.1$, $\gamma_R = 1$, $k_c = 0.1$, $k_t = 5$, $k_{RS} = 1500$, $\beta_N = 100$, $\beta_D =$ see function F2Dm(x), $\beta_R = 75$, $n = 1$ | |
| 2D, lower | 4–6 | $\gamma = 0.1$, $\gamma_R = 1$, $k_c = 10000$, $k_t = 5$, $k_{RS} = 1500$, $\beta_N = 100$, $\beta_D =$ see function F2Dl(x), $\beta_R = 75$, $p = 5$ | |
| 2E, upper | 1–3 | $\gamma = 0.1$, $\gamma_R = 1$, $k_c =$ as indicated, $k_t = 5$, $k_{RS} = 1500$, $\beta_N = 100$, $\beta_D =$ see function F2Eu(x), $\beta_R = 75$, $n = 1$ | |
| 2E, lower | 4–6 | $\gamma = 0.1$, $\gamma_R = 1$, $k_c = 10000$, $k_t = 5$, $k_{RS} = 1500$, $\beta_N = 100$, $\beta_D =$ see function F2El(x), $\beta_R = 75$, $p =$ as indicated | |
| 3A | 1–3 | $\gamma = 0.1$, $\gamma_R = 1$, $k_c = 0.1$, $k_t = 5$, $k_{RS} = 1500$, $\beta_N = 100$, $\beta_D =$ see function F3A(x), $\beta_R = 75$, $n = 1$ | |
| 3BC | 1–3, S1.5 | $\gamma = 0.1$, $\gamma_S = 1$, $k_c = 0.1$, $k_t = 5$, $\beta_N = 100$, $\beta_D = 160$ to 40 | |
| 4D | S1.3.4 | $\gamma = 1$, $\gamma_S = 1$, $\gamma_R = 1$, $k_t = 1$, $k_{DR} = 1$, $k_{RS} = 300000$, $\beta_N = 10$, $\beta_D = 100$, $\beta_R = 1000000$, $m = 1$, $n = 3$ | Cells initially set in a low DSL/high Notch condition with random perturbations from $D = \beta_D \cdot 10^{-5}$ and $N = \beta_N$ chosen uniformly from $\pm 10\%$. |
| 4E | S1.3.1 | $\gamma = 1$, $\gamma_S = 1$, $\gamma_R = 1$, $k_c = 0.1$, $k_t = 1$, $k_{DR} = 1$, $k_{RS} = 300000$, $\beta_N = 100$, $\beta_D = 10$, $\beta_R = 1000000$, $m = 1$, $n = 3$ | Cells initially set in a low DSL/high Notch condition with random perturbations from $D = \beta_D \cdot 10^{-5}$ and $N = \beta_N$ chosen uniformly from $\pm 10\%$. |

*continued on next page...*

*– continued*

| Figure | Equations | Parameters | Initial conditions and comments |
|---|---|---|---|
| 5AC | S1.3.4 | $\gamma = 1$, $\gamma_S = 1$, $\gamma_R = 1$, $k_c = 10000$, $k_t = 1$, $k_{DR} = 1$, $k_{RS} = 300000$, $\beta_N = 0.1$ to 1e5, $\beta_D = 0.1$ to 1e5, $\beta_R = 1000000$, $m = 1$, $n = 3$ (A) or 1 (C) | |
| 5BD | S1.3.1 | $\gamma = 1$, $\gamma_S = 1$, $\gamma_R = 1$, $k_c = 0.1$, $k_t = 1$, $k_{DR} = 1$, $k_{RS} = 300000$, $\beta_N = 0.1$ to 1e5, $\beta_D = 0.1$ to 1e5, $\beta_R = 1000000$, $m = 1$, $n = 3$ (B) or 1 (D) | |
| 6 | S1.3.2a,b | $\gamma = 1$, $\gamma_S = 1$, $k_c = 0.1$, $k_t = 1$, $k_{DR} = 1$,$k_{NS}$ = various, $\beta_N = 0.1$ to 1e4, $\beta_D = 0.01$ to 1e3, $m = 1$, $n = 1$, $\alpha_N = 0.01 \cdot \beta_N$ | |
| S1A | 6 | $p = 1$ to 10 | |
| S2 | 1–3 | $\gamma = 0.1$, $\gamma_R = 1$, $k_c = 0.1$, $k_t = 5$, $k_{RS} = 1500$, $\beta_N = 0.1$ to 3, $\beta_D$ = see function FS2(x), $\beta_R = 75$, $n = 1$ | |
| S3ACE | S1.3.4 | $\gamma = 1$, $\gamma_S = 1$, $\gamma_R = 1$, $k_c = 10000$, $k_t = 1$, $k_{DR} = 1$, $k_{RS} = 300000$, $\beta_N = 0.1$ to 1e5 (CE) or 10 (A), $\beta_D = 0.1$ to 1e5 (CE) or 50 (A), $\beta_R = 1000000$, $m = 1$, $n = 3$ | Cells initially set in a low DSL/high Notch condition with random perturbations from $D = \beta_D \cdot 10^{-5}$ and $N = \beta_N$ chosen uniformly from $\pm 10\%$. |
| S3BDF | S1.3.1 | $\gamma = 1$, $\gamma_S = 1$, $\gamma_R = 1$, $k_c = 0.1$, $k_t = 1$, $k_{DR} = 1$, $k_{RS} = 300000$, $\beta_N = 0.1$ to 1e5 (DF) or 10 (B), $\beta_D = 0.1$ to 1e5 (DF) or 50 (B), $\beta_R = 1000000$, $m = 1$, $n = 3$ | Cells initially set in a low DSL/high Notch condition with random perturbations from $D = \beta_D \cdot 10^{-5}$ and $N = \beta_N$ chosen uniformly from $\pm 10\%$. |
| S4ACE | S1.6.1 | $\alpha_{\mathrm{N}} = 0.1$ to 1e5 (CE) or 10 (A), $\gamma_{\mathrm{N}} = \gamma_{\mathrm{D}} = \gamma_{\mathrm{S}} = \gamma_{\mathrm{R}} = 1$, $k_c = $ 1e4, $k_t = 1$, $\beta_{\mathrm{mN}} = \beta_{\mathrm{mD}} = \beta_{\mathrm{m}} = \gamma_{\mathrm{mN}} = \gamma_{\mathrm{mD}} = \gamma_{\mathrm{m}} = 1$, $\alpha_{\mathrm{D}} = 0.1$ to 1e5 (CE) or 50 (A), $m$=1, $n$=3, $k_{RS} = 300000$, $\eta = 1$ (CE) or 1e4 (A) | For (E), cells initially set in a low DSL/high Notch condition with random perturbations from $D = \alpha_{\mathrm{D}} \cdot 10^{-5}$ and $N = \alpha_{\mathrm{N}}$ chosen uniformly from $\pm 10\%$. |
| S4BDF | S1.6.1 | $\alpha_{\mathrm{N}} = 0.1$ to 1e5 (DF) or 10 (B), $\gamma_{\mathrm{N}} = \gamma_{\mathrm{D}} = \gamma_{\mathrm{S}} = \gamma_{\mathrm{R}} = 1$, $k_c = 0.1$, $k_t = 1$, $\beta_{\mathrm{mN}} = \beta_{\mathrm{mD}} = \beta_{\mathrm{m}} = \gamma_{\mathrm{mN}} = \gamma_{\mathrm{mD}} = \gamma_{\mathrm{m}} = 1$, $\alpha_{\mathrm{D}} = 0.1$ to 1e5 (DF) or 50 (B), $m$=1, $n$=3, $k_{RS} = 300000$, $\eta = 1$ (DF) or 1e4 (B) | For (F), cells initially set in a low DSL/high Notch condition with random perturbations from $D = \alpha_{\mathrm{D}} \cdot 10^{-5}$ and $N = \alpha_{\mathrm{N}}$ chosen uniformly from $\pm 10\%$. |

$$\text{F2Dm}(x) = \beta_N \max\left(B\,|x| + 1 - 10.5B, 0\right) \text{ where } B = \{0.02, 0.05, 0.2\}$$

$$\text{F2Dl}(x) = \beta_N \max\left(B\,|x| + 0.5 - 10.5B, 0\right) \text{ where } B = \{0.02, 0.05, 0.2\}$$

$$\text{F2Eu}(x) = \beta_N \max\left(B\,|x| + 1 - 10.5B, 0\right) \text{ where } B = \{0.02 - 0.16\}$$

$$\text{F2El}(x) = \beta_N \max\left(B\,|x| + 0.5 - 10.5B, 0\right) \text{ where } B = \{0.02 - 0.16\}$$

$$\text{F3A}(x) = \beta_N \left(1 - \frac{f}{24}\,|x|\right) \text{ where } f = \{0.5 - 2.5\}$$

$$\text{FS2}(x) = \beta_N \left(1 - \frac{f}{24}\,|x|\right) \text{ where } f = \{0.3 - 10\}$$
