# Supplementary material for: Mutual Inactivation of Notch Receptors and Ligands Facilitates Developmental Patterning
Source: PLoS Comput Biol. 2011 Jun 9;7(6):e1002069. doi: 10.1371/journal.pcbi.1002069 (PMC3111533; doi:10.1371/journal.pcbi.1002069)
Supplement: Text S1 — Details of derivations mentioned in text. (PDF) [file pcbi.1002069.s006.pdf]

Supporting Information Text S1

S1.1: Derivation of equations governing Notch signaling

With inclusion of mutual inactivation (MI)

Notch signaling occurs via the *trans*-interaction of Notch (N_i) on the surface of a cell i with DSL (D_j) on the surfaces of its neighbors j, which initiates a sequence of biochemical events resulting in cleavage of the Notch receptor to free its intercellular signaling domain (S_i) for translocation to the nucleus, where it may induce the expression of some Notch signaling reporter (R_i). Additionally, Notch and DSL on the same cell surface (N_i and D_i) *cis*-inhibit by forming a complex that inactivates both molecules in what we term the Mutual Inactivation (MI) mechanism [1]. Thus the reactions we consider are the following:

$\mathrm{N}_i + \mathrm{D}_j \rightleftharpoons [\mathrm{N}_i\mathrm{D}_j] \rightarrow \mathrm{S}_i$	*trans*–activation, with association/dissociation rates $k_D^{\pm}$ and cleavage rate k_S
$\mathrm{N}_i + \mathrm{D}_i \rightleftharpoons [\mathrm{N}_i\mathrm{D}_i] \rightarrow \varnothing$	*cis*–inhibition, with association/dissociation rates $k_C^{\pm}$ and inactivation/dilution rate γ_{ND}
$\mathrm{S}_i \rightarrow \mathrm{R}_i$	Signal activation of reporter

The first reaction corresponds to *trans*-activation, the second to *cis*-inhibition with mutual inactivation, and the third to Notch signaling-mediated induction of reporter expression, as described above. These reactions are described by the following kinetic equations:

$$\dot{\mathrm{N}}_i = \alpha_{\mathrm{N}}\mathrm{m}_{\mathrm{N}_i} - \gamma_{\mathrm{N}}\mathrm{N}_i - \left(k_{\mathrm{D}}^{+} \sum_{j=]i[} \frac{1}{l_{ij}} \mathrm{N}_i\mathrm{D}_j - k_{\mathrm{D}}^{-} \sum_{j=]i[} \frac{1}{l_{ij}} [\mathrm{N}_i\mathrm{D}_j] \right) - \left(k_{\mathrm{C}}^{+}\mathrm{N}_i\mathrm{D}_i - k_{\mathrm{C}}^{-} [\mathrm{N}_i\mathrm{D}_i] \right) \tag{S1.1.1a}$$

$$\dot{\mathrm{m}}_{\mathrm{N}_i} = \eta \left(\beta_{\mathrm{mN}} - \gamma_{\mathrm{mN}}\mathrm{m}_{\mathrm{N}_i} \right) \tag{S1.1.1b}$$

$$\dot{\mathrm{D}}_i = \alpha_{\mathrm{D}}\mathrm{m}_{\mathrm{D}_i} - \gamma_{\mathrm{D}}\mathrm{D}_i - \left(k_{\mathrm{D}}^{+} \sum_{j=]i[} \frac{1}{l_{ij}} \mathrm{N}_j\mathrm{D}_i - k_{\mathrm{D}}^{-} \sum_{j=]i[} \frac{1}{l_{ij}} [\mathrm{N}_j\mathrm{D}_i] \right) - \left(k_{\mathrm{C}}^{+}\mathrm{N}_i\mathrm{D}_i - k_{\mathrm{C}}^{-} [\mathrm{N}_i\mathrm{D}_i] \right) \tag{S1.1.1c}$$

$$\dot{\mathrm{m}}_{\mathrm{D}_i} = \eta \left(\beta_{\mathrm{mD}} - \gamma_{\mathrm{mD}}\mathrm{m}_{\mathrm{D}_i} \right) \tag{S1.1.1d}$$

$$[\mathrm{N}_i\dot{\mathrm{D}}_j] = k_{\mathrm{D}}^{+}\mathrm{N}_i\mathrm{D}_j - k_{\mathrm{D}}^{-} [\mathrm{N}_i\mathrm{D}_j] - k_{\mathrm{S}} [\mathrm{N}_i\mathrm{D}_j] \tag{S1.1.1e}$$

$$[\mathrm{N}_i\dot{\mathrm{D}}_i] = k_{\mathrm{C}}^{+}\mathrm{N}_i\mathrm{D}_i - k_{\mathrm{C}}^{-} [\mathrm{N}_i\mathrm{D}_i] - \gamma_{ND} [\mathrm{N}_i\mathrm{D}_i] \tag{S1.1.1f}$$

$$\dot{\mathrm{S}}_i = k_{\mathrm{S}} \sum_{j=]i[} \frac{1}{l_{ij}} [\mathrm{N}_i\mathrm{D}_j] - \gamma_{\mathrm{S}}\mathrm{S}_i \tag{S1.1.1g}$$

$$\dot{\mathrm{m}}_{\mathrm{R}i} = \eta \left(f_A \left(\mathrm{S}_i; \beta_{\mathrm{m}}, n, k_{\mathrm{RS}} \right) - \gamma_{\mathrm{m}}\mathrm{m}_{\mathrm{R}i} \right) \tag{S1.1.1h}$$

$$\dot{\mathrm{R}}_i = \alpha_R\mathrm{m}_{\mathrm{R}i} - \gamma_{\mathrm{R}}\mathrm{R}_i \tag{S1.1.1i}$$

The notation $j =]i[$ refers to indices j representing neighbors of cell i and l_{ij} measures the ratio of the length of the interface between cells i and j and the total perimeter of cell i, reflecting the assumption that Notch and

DSL are uniformly distributed on the cell surface. The increasing Hill function $f_A(\mathrm{S}_i;\beta_\mathrm{m},n,k_\mathrm{RS}) \equiv \beta_m \frac{\mathrm{S}_i^n}{k_\mathrm{RS}+\mathrm{S}_i^n}$ phenomenologically parametrizes the transcriptional promotion process. The parameter η scales the dynamics of the mRNA molecules while preserving their steady-state values. We work mostly in the regime of γ_S, k_S, γ_ND, and η large, where the dynamics of the *trans* intermediate $[\mathrm{N}_i\mathrm{D}_j]$, intracellular signal S_i, and the mRNA are rapid relative to the Notch/DSL and Reporter protein dynamics, allowing the quasi-steady-state approximation to their dynamics ($[\dot{\mathrm{N}_i\mathrm{D}_j}] \approx \dot{\mathrm{S}}_i \approx \dot{m}_{\mathrm{X}_i} \approx 0$). These approximations are made here for convenience of presentation, and relaxing them does not modify our conclusions (Section 5). In particular, Fig. S4 demonstrates this for mRNA dynamics on a scale comparable to the first-order Notch and DSL protein lifetimes ($\eta = 1$ with $\gamma_\mathrm{mN} = \gamma_\mathrm{mD} = \gamma_\mathrm{mR} = 1$). Furthermore, we assume that Notch binds to *cis*-Delta irreversibly ($k_\mathrm{C}^- = 0$), and thus Notch dynamics become independent of the $[\mathrm{N}_i\mathrm{D}_i]$ complex. With these approximations, the model is reduced to

$$\dot{\mathrm{N}}_i = \beta_\mathrm{N} - \gamma\mathrm{N}_i - \mathrm{N}_i\frac{\langle D_j\rangle_i}{k_t} - \mathrm{N}_i\frac{\mathrm{D}_i}{k_c} \quad \text{(S1.1.2a)}$$

$$\dot{\mathrm{D}}_i = \beta_\mathrm{D} - \gamma\mathrm{D}_i - \frac{\langle \mathrm{N}_j\rangle_i}{k_t}\mathrm{D}_i - \mathrm{N}_i\frac{\mathrm{D}_i}{k_c} \quad \text{(S1.1.2b)}$$

$$\dot{\mathrm{R}}_i = f_A\left(\frac{1}{\gamma_\mathrm{S}}\mathrm{N}_i\frac{\langle D_j\rangle_i}{k_t};\beta_\mathrm{R},n,k_\mathrm{RS}\right) - \gamma_\mathrm{R}\mathrm{R}_i \quad \text{(S1.1.2c)}$$

where we have defined $\beta_\mathrm{N} = \frac{\beta_\mathrm{mN}\alpha_N}{\gamma_\mathrm{mN}}$,$\beta_\mathrm{D} = \frac{\beta_\mathrm{mD}\alpha_D}{\gamma_\mathrm{mD}}$,$\beta_\mathrm{R} = \frac{\beta_\mathrm{m}\alpha_R}{\gamma_\mathrm{m}}$, $k_t^{-1} \equiv \frac{k_\mathrm{D}^+k_\mathrm{S}}{k_\mathrm{D}^-+k_\mathrm{S}}$ and $k_c^{-1} \equiv k_\mathrm{C}^+$, and employed the notation $\langle X_j\rangle_i \equiv \sum_{j=]i[}\frac{1}{l_{ij}}X_j$ for the average of the enclosed quantity in the neighbors j of cell i weighted by the magnitudes of the cell-cell interfaces. We have also taken the simplifying assumption that $\gamma_\mathrm{N} = \gamma_\mathrm{D} \equiv \gamma$. Solving with different degradation rates is straightforward, and is guaranteed to leave conclusions relating to the system's steady-state properties unchanged (as is clear from the freedom to rescale parameters).

Excluding *cis*–inhibition

By reviewing the preceding derivation and omitting those terms relating to *cis*-inhibition, we see that the kinetic equations become

$$\dot{\mathrm{N}}_i = \beta_\mathrm{N} - \gamma\mathrm{N}_i - \mathrm{N}_i\frac{\langle \mathrm{D}_j\rangle_i}{k_t} \quad \text{(S1.1.3a)}$$

$$\dot{\mathrm{D}}_i = \beta_\mathrm{D} - \gamma\mathrm{D}_i - \frac{\langle \mathrm{N}_j\rangle_i}{k_t}\mathrm{D}_i \quad \text{(S1.1.3b)}$$

$$\dot{\mathrm{S}}_i = \mathrm{N}_i\frac{\langle \mathrm{D}_j\rangle_i}{k_t} - \gamma_\mathrm{S}\mathrm{S}_i \approx 0 \rightarrow \mathrm{S}_i \approx \frac{1}{\gamma_\mathrm{S}}\mathrm{N}_i\frac{\langle \mathrm{D}_j\rangle_i}{k_t} \quad \text{(S1.1.3c)}$$

$$\dot{\mathrm{R}}_i = f_A\left(\frac{1}{\gamma_\mathrm{S}}\mathrm{N}_i\frac{\langle D_j\rangle_i}{k_t};\beta_\mathrm{R},n,k_\mathrm{RS}\right) - \gamma_\mathrm{R}\mathrm{R}_i \quad \text{(S1.1.3d)}$$

S1.2: Boundary formation

With mutual inactivation

If the expression rate of DSL varies spatially (i.e. $\beta_{\rm D} \rightarrow \beta_{\rm D}(x)$) the dynamics of Notch signaling with MI are governed by the following equations:

$$\dot{\rm N}_i = \beta_{\rm N} - \gamma {\rm N}_i - {\rm N}_i \frac{\langle D_j \rangle_i}{k_t} - {\rm N}_i \frac{{\rm D}_i}{k_c} \tag{S1.2.1a}$$

$$\dot{\rm D}_i = \beta_{\rm D}\left(x\right) - \gamma {\rm D}_i - \frac{\langle {\rm N}_j \rangle_i}{k_t} {\rm D}_i - {\rm N}_i \frac{{\rm D}_i}{k_c} \tag{S1.2.1b}$$

$$\dot{\rm R}_i = f_A\left(\frac{1}{\gamma_{\rm S}} {\rm N}_i \frac{\langle D_j \rangle_i}{k_t}; \beta_{\rm R}, n, k_{\rm RS}\right) - \gamma_{\rm R} {\rm R}_i \tag{S1.2.1c}$$

These equations, labeled (1)–(3) in the main paper text, are sufficient to generate sharply-defined bands of Notch signaling at the crossing point (supposing its existence) between the DSL and Notch expression rates.

Without *cis*–inhibition — The Band–Pass Filter model

In the absence of *cis*–inhibition, a mechanism that explicitly limits the report of Notch signaling to a band of signaling levels is required for the conversion of a DSL expression gradient to strips of signal Reporter activity. The band–pass model described in the main text is governed by a modification of the equations (S1.1.3a)–(S1.1.3d) to allow for spatially-varying DSL expression and restrict Reporter expression to a narrow band of Signal induction levels, yielding the following equations:

$$\dot{\rm N}_i = \beta_{\rm N} - \gamma {\rm N}_i - {\rm N}_i \frac{\langle {\rm D}_j \rangle_i}{k_t} \tag{S1.2.2a}$$

$$\dot{\rm D}_i = \beta_{\rm D}\left(x\right) - \gamma {\rm D}_i - \frac{\langle {\rm N}_j \rangle_i}{k_t} {\rm D}_i \tag{S1.2.2b}$$

$$\dot{\rm S}_i = {\rm N}_i \frac{\langle {\rm D}_j \rangle_i}{k_t} - \gamma_{\rm S} {\rm S}_i \approx 0 \rightarrow {\rm S}_i \approx \frac{1}{\gamma_{\rm S}} {\rm N}_i \frac{\langle {\rm D}_j \rangle_i}{k_t} \tag{S1.2.2c}$$

$$\dot{\rm R}_i = \beta_{\rm R} \frac{{\rm S}_i^p}{k_{\rm b}^p + {\rm S}_i^p} \frac{k_{\rm b}^q}{k_{\rm b}^q + {\rm S}_i^q} - \gamma_{\rm R} {\rm R}_i = \beta_{\rm R} \frac{\left({\rm N}_i \langle {\rm D}_j \rangle_i\right)^p}{k_{\rm RS}^p + \left({\rm N}_i \langle {\rm D}_j \rangle_i\right)^p} \frac{k_{\rm RS}^p}{k_{\rm RS}^p + \left({\rm N}_i \langle {\rm D}_j \rangle_i\right)^p} - \gamma_{\rm R} {\rm R}_i \tag{S1.2.2d}$$

corresponding to equations (4)–(6) in the main text.

S1.3: Lateral inhibition

Transcriptional lateral inhibition with mutual inactivation (LIMI)

With the condition that the production rate of DSL may be repressed by the reporter ${\rm R}_i$, i.e. $\beta_{\rm D} \rightarrow f_R\left({\rm R}_i; \beta_{\rm D}, m, k_{\rm DR}\right)$ where $f_R\left({\rm R}_i; \beta_{\rm D}, m, k_{\rm DR}\right) \equiv \beta_{\rm D} \frac{k_{\rm DR}}{k_{\rm DR} + {\rm R}_i^m}$ is a repressive Hill function, we have the equations representing lateral inhibition by transcriptional downregulation of DSL with mutual inactivation. It is convenient to convert the equations to a set of dimensionless parameters as follows: $t \equiv \gamma_R {\rm t}$, $N \equiv \frac{\rm N}{N_0}$, $D \equiv \frac{\rm D}{D_0}$, and $R \equiv \frac{\rm R}{R_0}$ where $N_0 = D_0 \equiv \gamma k_t$,

and $R_0 \equiv k_{\mathrm{DR}}$. The equations are then

$$\tau \dot{N}_i = \beta_N - N_i - N_i \left\langle D_j \right\rangle_i - N_i \frac{D_i}{\kappa_c} \tag{S1.3.1a}$$

$$\tau \dot{D}_i = f_R\left(R_i; \beta_D, m, 1\right) - D_i - \left\langle N_j \right\rangle_i D_i - N_i \frac{D_i}{\kappa_c} \tag{S1.3.1b}$$

$$\dot{R}_i = f_A\left(N_i \left\langle D_j \right\rangle_i; \beta_R, n, k_{RS}\right) - R_i \tag{S1.3.1c}$$

where $\tau \equiv \frac{\gamma_{\mathrm{R}}}{\gamma}$, $\beta_N \equiv \frac{\beta_{\mathrm{N}}}{\gamma N_0}$, $\beta_D \equiv \frac{\beta_{\mathrm{D}}}{\gamma D_0}$, $\beta_R \equiv \frac{\beta_{\mathrm{R}}}{\gamma_{\mathrm{R}} R_0}$, $\kappa_c \equiv \frac{k_c}{k_t}$, and $k_{RS} \equiv \frac{k_{\mathrm{RS}}\gamma_{\mathrm{S}} k_t}{N_0 D_0}$. These correspond to the equations labeled (10)–(12) in the main text.

Simplest lateral inhibition with mutual inactivation (SLIMI)

The mutual inactivation mechanism permits a lateral inhibition mechanism driven by a single feedback connecting Notch expression to Notch signaling, as follows:

$$\dot{\mathrm{N}}_i = \alpha_{\mathrm{N}} + f_A\left(\frac{1}{\gamma_{\mathrm{S}}} \mathrm{N}_i \frac{\left\langle \mathrm{D}_j \right\rangle_i}{k_t}; \beta_{\mathrm{N}}, n, k_{\mathrm{NS}}\right) - \gamma \mathrm{N}_i - \mathrm{N}_i \frac{\left\langle \mathrm{D}_j \right\rangle_i}{k_t} - \mathrm{N}_i \frac{\mathrm{D}_i}{k_c} \tag{S1.3.2a}$$

$$\dot{\mathrm{D}}_i = \beta_{\mathrm{D}} - \gamma \mathrm{D}_i - \frac{\left\langle \mathrm{N}_j \right\rangle_i}{k_t} \mathrm{D}_i - \mathrm{N}_i \frac{\mathrm{D}_i}{k_c} \tag{S1.3.2b}$$

where we have included a promoter "leakiness" term (α_{N}, representing imperfect repression) in the kinetic equation for the regulated component, which in this case is Notch. Here we use a set of dimensionless parameters as follows: $t \equiv \gamma \mathrm{t}$, $N \equiv \frac{\mathrm{N}}{N_0}$, and $D \equiv \frac{\mathrm{D}}{D_0}$ where $N_0 = D_0 \equiv \gamma k_t$

$$\dot{N}_i = \alpha_N + f_A\left(N_i \left\langle D_i \right\rangle_j; \beta_N, n, k_{NS}\right) - N_i - N_i \left\langle D_j \right\rangle_i - N_i \frac{D_i}{\kappa_c} \tag{S1.3.3a}$$

$$\dot{D}_i = \beta_D - D_i - \left\langle N_j \right\rangle_i D_i - N_i \frac{D_i}{\kappa_c} \tag{S1.3.3b}$$

where we have defined $\alpha_N \equiv \frac{\alpha_{\mathrm{N}}}{\gamma N_0}$, $\beta_N \equiv \frac{\beta_{\mathrm{N}}}{\gamma N_0}$, $\beta_D \equiv \frac{\beta_{\mathrm{D}}}{\gamma D_0}$, $\kappa_c \equiv \frac{k_c}{k_t}$, and $k_{NS} \equiv \frac{k_{\mathrm{NS}}\gamma_{\mathrm{S}} k_t}{N_0 D_0}$. These equations are used in Fig. 6.

Without *cis*-inhibition (LI)

With the condition that the production rate of DSL may be repressed by the reporter R_i, i.e. $\beta_{\mathrm{D}} \to \beta_{\mathrm{D}} \frac{k_{\mathrm{DR}}}{k_{\mathrm{DR}} + \mathrm{R}_i^m}$, combined with equations (S1.1.3a)–(S1.1.3d), we have the equations representing "canonical" lateral inhibition by transcriptional downregulation of DSL. It is convenient to convert the equations transforming variables as $t \equiv \mathrm{t}\gamma_{\mathrm{R}}$, $N \equiv \frac{\mathrm{N}}{N_0}$, $D \equiv \frac{\mathrm{D}}{D_0}$, and $R \equiv \frac{\mathrm{R}}{k_{\mathrm{DR}}}$ where $N_0 \equiv \frac{\beta_{\mathrm{N}}}{\gamma}$ and $D_0 \equiv \frac{\gamma_{\mathrm{S}} k_{\mathrm{RS}}}{k_t} \frac{1}{N_0}$ to give

$$\tau \dot{N}_i = 1 - N_i - N_i \left\langle D_j \right\rangle_i \tag{S1.3.4a}$$

$$\tau \dot{D}_i = f_R\left(R_i; \beta_D, m, 1\right) - D_i - \left\langle N_j \right\rangle_i D_i \tag{S1.3.4b}$$

$$\dot{R}_i = f_A\left(N_i \left\langle D_j \right\rangle_i; \beta_R, n, 1\right) - R_i \tag{S1.3.4c}$$

where $\tau \equiv \frac{\gamma_{\mathrm{R}}}{\gamma}$, $\beta_D \equiv \frac{\beta_{\mathrm{D}}}{D_0 \gamma}$, and $\beta_R \equiv \frac{\beta_{\mathrm{R}}}{k_{\mathrm{DR}} \gamma_{\mathrm{R}}}$. These correspond to the kinetic equations governing the system of which certain properties are plotted in Figs. 4D, 5AC of the main text and S3ACE of the Supporting Information.

S1.4: Linear stability analysis of lateral inhibition equations

It is immediately clear that a necessary condition for spontaneous development of a lateral inhibition pattern from an initially near-homogeneous collection of cells is the instability of the homogeneous steady state (N^*, D^*, R^*) in which every cell has the same value of N_i, D_i, and R_i. Thus a linear stability analysis about the homogeneous steady state can provide necessary conditions for patterning [2]. The stability analysis requires the computation of the Jacobian at the homogeneous steady state, which is in this case complicated by the large number of variables (three times the number of cells). This is made simpler by an observation originally from Othmer and Scriven [3] that the Jacobian can be expressed as the sum of two tensor products of matrices, one for the internal dynamics and the other for interactions with neighbors: $J = I_k \otimes H + M \otimes B$. The matrix tensor product is defined as $A \otimes B = \begin{pmatrix} a_{11}B & \cdots & a_{1k}B \\ \vdots & \ddots & \vdots \\ a_{k1}B & \cdots & a_{kk}B \end{pmatrix}$. Also, here I_k is the $k \times k$ identity matrix (k is the number of cells involved in the interactions in question), $H_{ij} = \frac{\partial \dot{q}_i}{\partial q_j}$ is the change in production of species i for a change in species j in the same cell, $B_{ij} = \frac{\partial \dot{q}_i}{\partial \langle q_j \rangle}$ is the change in production of species i for a change in species j in a neighboring cell, and M is the connectivity matrix defined as $M_{ij} = \begin{cases} 1/6 & \text{if } i \text{ and } j \text{ are neighbors} \\ 0 & \text{otherwise} \end{cases}$. Notch, Delta, and Reporter correspond to species $i = 1, 2, 3$ respectively.

Once the Jacobian has been written in this form, Othmer and Scriven further show that its eigenvalues are the eigenvalues of the various matrices $H + q_k B$ where q_k are the eigenvalues of the connectivity matrix M. An analysis of the matrix M in [3] tells us that $q_k \geq -0.5$, meaning that we need only compute an eigenvalue for the extreme case $q_k = -0.5$ to determine if the highest eigenvalue (known as the Maximum Lyapunov Exponent — MLE) has a positive real part, simplifying the problem enormously. We can execute this process for each of the lateral inhibition models we have described above to compute their MLE profiles as a function of various parameters, as plotted in Figs. 5 and 6. The derivations of the MLEs are as follows:

Relevant partial derivatives

i) "Canonical" LI

$$H = \frac{1}{\tau}\begin{pmatrix} -1 - \langle D_j \rangle_i & 0 & 0 \\ 0 & -1 - \langle N_j \rangle_i & -\frac{m}{\beta_D R_i} f_A\left(R_i; \beta_D, m, 1\right) f_R\left(R_i; \beta_D, m, 1\right) \\ \tau \frac{n}{\beta_R N_i} f_A\left(N_i \langle D_j \rangle_i ; \beta_R, n, 1\right) f_R\left(N_i \langle D_j \rangle_i ; \beta_R, n, 1\right) & 0 & -\tau \end{pmatrix}$$

$$B = \frac{1}{\tau}\begin{pmatrix} 0 & -N_i & 0 \\ -D_i & 0 & 0 \\ 0 & \frac{n\tau}{\beta_R D_j} f_A\left(S_i; \beta_R, n, 1\right) f_R\left(S_i; \beta_R, n, 1\right) & 0 \end{pmatrix}$$

ii) LIMI

$$H = \frac{1}{\tau}\begin{pmatrix} -\left(1 + \langle D_j \rangle_i + \frac{D_i}{\kappa_c}\right) & -\frac{N_i}{\kappa_c} & 0 \\ -\frac{D_i}{\kappa_c} & -\left(1 + \langle N_j \rangle_i + \frac{N_i}{\kappa_c}\right) & -\frac{m}{\beta_D R_i} f_A\left(R_i; \beta_D, m, 1\right) f_R\left(R_i; \beta_D, m, 1\right) \\ \frac{n\tau}{\beta_R N_i} f_A\left(S_i; \beta_R, n, k_{RS}\right) f_R\left(S_i; \beta_R, n, k_{RS}\right) & 0 & -\tau \end{pmatrix}$$

$$B = \frac{1}{\tau}\begin{pmatrix} 0 & -N_i & 0 \\ -D_i & 0 & 0 \\ 0 & \frac{n\tau}{\beta_R D_j} f_A\left(S_i;\beta_R,n,k_{RS}\right) f_R\left(S_i;\beta_R,n,k_{RS}\right) & 0 \end{pmatrix}$$

iii) SLIMI

$$H = \begin{pmatrix} \frac{n}{\beta_N N_i} f_A\left(S_i;\beta_N,n,k_{NS}\right) f_R\left(S_i;\beta_N,n,k_{NS}\right) - \left(1 + \left\langle D_j \right\rangle_i + \frac{D_i}{\kappa_c}\right) & -\frac{N_i}{\kappa_c} \\ -\frac{D_i}{\kappa_c} & -\left(1 + \left\langle N_j \right\rangle_i + \frac{N_i}{\kappa_c}\right) \end{pmatrix}$$

$$B = \begin{pmatrix} 0 & \frac{n}{\beta_N D_j} f_A\left(S_i;\beta_N,n,k_{NS}\right) f_R\left(S_i;\beta_N,n,k_{NS}\right) - N_i \\ -D_i & 0 \end{pmatrix}$$

Evaluation of the homogeneous steady state

In each case the homogeneous steady state of the system was found numerically by solving the systems of equations for $N_i = N$, $D_i = D$, and (if relevant) $R_i = R$.

Diagonalization of the reduced Jacobian

The matrices H and B, evaluated at the values N, D, and R fixed by the homogeneous steady state, were combined as prescribed in [3] and diagonalized with $q_k = -0.5$ which is the extreme eigenvalue of the structure matrix M for a regular hexagonal lattice. The diagonalization may be written explicitly in terms of the homogeneous steady state values and q_k in each case because the characteristic equation is of order three or less, but the expressions are complicated and not very illuminating. The maximal resulting eigenvalue is the MLE.

S1.5: Noise in boundary formation

As written in the main text, based on an intuitive understanding of the mutual inactivation mechanism we suspect that MI-based models might be more sensitive to intrinsic sources of noise (contributing to uncorrelated variabilities of Notch and DSL production in a given cell) than those that are extrinsic (by which the Notch and DSL production rate variabilities in a given cell are correlated). To test this we numerically simulate the boundary formation process subject to static noise in the Notch and Delta production rates with varying degrees of correlation between their variability, ranging from fully-intrinsic (correlation coefficient = 0) to fully-extrinsic (correlation coefficient = 1). In order to make the comparisons in the outcome of interest (the variability in the location of the vein boundary-defining peak) fairly we must also be able to control the total variability in the Notch and DSL production rates *independently* of the correlation between them.

Using a model of multiplicative noise, in which the production rates of Notch in each cell are $\beta_{N,i} = \xi_N \left\langle \beta_N \right\rangle$ and the production rates of DSL are $\beta_{D,i} = \xi_D \left\langle \beta_D \right\rangle$, we thus seek to draw the random variables ξ_N and ξ_D such that:

1. Means are preserved, with $\left\langle \xi_N \right\rangle = \left\langle \xi_D \right\rangle = 1$
2. Standard deviations are equal and set to some arbitrary σ, with $\sigma_{\xi_N} = \sigma_{\xi_D} = \sigma$

3. The correlation between the variations in each production rate is some arbitrarily chosen r between zero and one, with $r_{\xi_N \xi_D} = r$

4. Unphysical negative production rates are excluded, with $\xi_N, \xi_D \geq 0$.

We have chosen a mechanism of achieving this that entails choosing two uncorrelated random variables x and y from normal distributions of mean zero and standard deviations σ_x and σ_y, respectively, rotating x and y by an angle θ and shifting the result by Λ in each direction to generate distributions u and v, and then exponentiating each to generate the final distributions ξ_{β_N} and ξ_{β_D}. The required conditions fix the free parameters σ_x, σ_y, θ, and Λ as follows:

The distributions u and v are drawn from $u = x\cos\theta - y\sin\theta + \Lambda$ and $v = x\sin\theta + y\cos\theta + \Lambda$, from which we have that $\langle u \rangle = \langle v \rangle = \Lambda$, $\sigma_u^2 = \sigma_x^2 \cos^2\theta + \sigma_y^2 \sin^2\theta$, and $\sigma_v^2 = \sigma_x^2 \sin^2\theta + \sigma_y^2 \cos^2\theta$. Then the distributions ξ_N and ξ_D drawn from $\xi_N = e^u$ and $\xi_D = e^v$ yield $\langle \xi_N \rangle = e^{\Lambda + \frac{1}{2}\sigma_u^2}$, $\langle \xi_D \rangle = e^{\Lambda + \frac{1}{2}\sigma_v^2}$, $\sigma_{\xi_N}^2 = \langle \xi_N \rangle^2 \left(e^{\sigma_u^2} - 1\right)$, and $\sigma_{\xi_D}^2 = \langle \xi_D \rangle^2 \left(e^{\sigma_v^2} - 1\right)$. By the first requirement that the averages of the random variables ξ must be equal to one, we have that $\sigma_u = \sigma_v \rightarrow \theta = \frac{\pi}{4}$, and that $1 = e^{\Lambda + \frac{1}{2}\sigma_u^2} \rightarrow \Lambda = -\frac{1}{2}\sigma_u^2$. The second requirement provides $\sigma^2 = e^{\sigma_u^2} - 1 \rightarrow \sigma_u^2 = \ln\left(\sigma^2 + 1\right) \rightarrow \Lambda = -\frac{1}{2}\ln\left(\sigma^2 + 1\right)$. The final condition $r_{\xi_N \xi_D} = r$ provides $\sigma^2 r + 1 = \langle \xi_N \xi_D \rangle$. By computing $\langle \xi_N \xi_D \rangle = \langle e^{u+v} \rangle = e^{2\Lambda} \left\langle e^{\sqrt{2}x} \right\rangle = e^{2\Lambda + \sigma_x^2}$ this provides $\sigma_x^2 = \ln\left(\left(\sigma^2 r + 1\right)\left(\sigma^2 + 1\right)\right)$ and correspondingly $\sigma_y^2 = \ln\left(\frac{\sigma^2+1}{r\sigma^2+1}\right)$.

Thus the following algorithm generates positive random distributions ξ_N and ξ_D such that their means are one, standard deviations are σ, and correlation coefficient is r:

1. Draw from two independent normal distributions x and y with means zero and standard deviations $\sigma_x = \sqrt{\ln\left(\left(\sigma^2 r + 1\right)\left(\sigma^2 + 1\right)\right)}$ and $\sigma_y = \sqrt{\ln\left(\frac{\sigma^2+1}{r\sigma^2+1}\right)}$

2. From these, generate two related random distributions $u = \frac{1}{\sqrt{2}}(x - y) - \frac{1}{2}\ln\left(\sigma^2 + 1\right)$ and $v = \frac{1}{\sqrt{2}}(x + y) - \frac{1}{2}\ln\left(\sigma^2 + 1\right)$

3. Let ξ_N be drawn from e^u and ξ_D be drawn from e^v

S1.6: Influence of finite Signal and mRNA lifetimes

In the analyses described above, our calculations of the MLE explicitly neglected the dynamics of signaling intermediates and mRNA molecules. This formally describes the limit in which the dynamics of those components are assumed to be inifinitely fast, or at least much faster than the timescales on which the included components change (namely, the protein degradation rates). Such an assumption is perhaps uncomfortably strong, especially with respect to mRNA dynamics. We have therefore considered the effect of finite mRNA lifetimes in lateral inhibition

patterning by explicitly modeling the entire system according to the following equations:

$$\dot{\mathrm{N}}_i = \alpha_{\mathrm{N}}\mathrm{m}_{\mathrm{N}_i} - \gamma_{\mathrm{N}}\mathrm{N}_i - \frac{1}{k_c}\mathrm{N}_i\mathrm{D}_i - \frac{1}{k_t}\mathrm{N}_i\left\langle \mathrm{D}_j \right\rangle_i \tag{S1.6.1a}$$

$$\dot{\mathrm{m}}_{\mathrm{N}_i} = \eta\left(\beta_{\mathrm{mN}} - \gamma_{\mathrm{mN}}\mathrm{m}_{\mathrm{N}_i}\right) \tag{S1.6.1b}$$

$$\dot{\mathrm{D}}_i = \alpha_{\mathrm{D}}\mathrm{m}_{\mathrm{D}_i} - \gamma_{\mathrm{D}}\mathrm{D}_i - \frac{1}{k_c}\mathrm{N}_i\mathrm{D}_i - \frac{1}{k_t}\left\langle \mathrm{N}_j \right\rangle_i \mathrm{D}_i \tag{S1.6.1c}$$

$$\dot{\mathrm{m}}_{\mathrm{D}_i} = \eta\left(\beta_{\mathrm{mD}}\frac{1}{1+\mathrm{R}_i^m} - \gamma_{\mathrm{mD}}\mathrm{m}_{\mathrm{D}_i}\right) \tag{S1.6.1d}$$

$$\dot{\mathrm{S}}_i = \frac{1}{k_t}N_i\left\langle D_j \right\rangle_i - \gamma_{\mathrm{S}}\mathrm{S}_i \tag{S1.6.1e}$$

$$\dot{\mathrm{m}}_{\mathrm{R}i} = \eta\left(f_A\left(\mathrm{S}_i;\beta_{\mathrm{m}},n,k_{\mathrm{RS}}\right) - \gamma_{\mathrm{m}}\mathrm{m}_{\mathrm{R}i}\right) \tag{S1.6.1f}$$

$$\dot{\mathrm{R}}_i = \alpha_R\mathrm{m}_{\mathrm{R}i} - \gamma_{\mathrm{R}}\mathrm{R}_i \tag{S1.6.1g}$$

These equations with very large k_c represent the LI model. In the MLE calculation, we find that including mRNA dynamics on a timescale comparable to those of the proteins ($\eta = 1$ with $\gamma_{\mathrm{mN}} = \gamma_{\mathrm{mD}} = \gamma_{\mathrm{mR}} = 1$) decreases the magnitude of the real part of the MLE, but does not change its sign (Fig. S4ABCD). Our conclusions regarding the stability of the homogeneous steady state, and therefore the tendency of the system to pattern, are thus unaffected.

With respect to the dynamical simulations, incorporating finite mRNA lifetimes slows the overall process, but our conclusion regarding the effect of the MI interaction predominantly decreasing the homogeneous patterning time is unchanged (Fig. S4EF).

References

[1] Sprinzak D et al. (2010) *Cis*-interactions between Notch and Delta generate mutually exclusive signalling states. *Nature* 465:86–90.

[2] Plahte E. (2001) Pattern formation in discrete cell lattices. *J Math Biol* 43:411–445.

[3] Othmer HG, Scriven LE. (1971) Instability and dynamic pattern in cellular networks. *J Theor Biol* 32:507–537.
